# The H3K9me2-FOXG1-microRNA axis reduces cochlear hair cells damage by modulating autophagy in age-related hearing loss

**DOI:** 10.3389/fnmol.2026.1834102

**Published:** 2026-05-08

**Authors:** Sihui Wen, Yongping Huang, Yurong Mu, Caini Li, Bowen Xu, Shengyu Zou, Chunjiang Wei, Kun Lin, Tingting Wu, Peng Zhang, Zuhong He

**Affiliations:** 1 Department of Otorhinolaryngology-Head and Neck Surgery, Zhongnan Hospital of Wuhan University, Wuhan, China; 2 Hubei Key Laboratory of Immunology and Metabolism Research in Otolaryngology Diseases, Wuhan, China; 3 Taikang Medical School (School of Basic Medical Sciences), Wuhan University, Wuhan, China; 4 Department of Otolaryngology-Head and Neck Surgery, Shandong Provincial ENT Hospital, Shandong University, Jinan, China; 5 Shandong Key Laboratory of Deafness and Vertigo, Shandong Key Laboratory of Vertigo and Vestibular Medicine, Jinan Key Laboratory of Vertigo and Balance Medicine, Shandong Clinical Medical Research Center for Otolaryngological Diseases, Shandong Second Provincial General Hospital, Shandong Institute of Otorhinolaryngology, Jinan, China; 6 Department of Otolaryngology Head and Neck Surgery, Zhongda Hospital, School of Life Sciences and Technology, Advanced Institute for Life and Health, Nanjing, China

**Keywords:** age-related hearing loss, autophagy, FOXG1, H3K9me2, hair cells, microRNAs

## Abstract

Age-related hearing loss (ARHL) is a growing global health concern due to its irreversibility and multifactorial pathogenesis. Epigenetic alterations are emerging as key drivers of aging, yet the mechanisms underlying their contribution to ARHL remain poorly understood. Here, we identified the histone H3 lysine 9 dimethylation (H3K9me2)-forkhead box G1 (FOXG1)-microRNA axis as a crucial regulator of auditory degeneration through modulation of autophagy. Using *in vivo* and *in vitro* D-galactose-induced aging models, we observed that H3K9me2 levels exhibited an inverse relationship with FOXG1 expression in cochlear hair cells. FOXG1 regulated autophagy by controlling autophagy-related microRNAs, thereby modulating reactive oxygen species accumulation and apoptosis in aging hair cells. Furthermore, a multi-omics approach delineated the broader FOXG1-mediated regulatory network driving ARHL. To our knowledge, this is the first study to comprehensively characterize the epigenetic regulation of autophagy by FOXG1 in ARHL, providing new mechanistic insights into cochlear hair cell aging and potential therapeutic targets.

## 1 Introduction

Presbycusis, or age-related hearing loss (ARHL) (Ninoyu and Friedman, 2024), represents a major healthcare and social challenge in rapidly aging populations. ARHL significantly impairs communication, quality of life, and psychological wellbeing among older adults (Livingston et al., 2020). It typically manifests as a progressive, irreversible, bilateral, and symmetric sensorineural hearing loss (He Z. H. et al., 2019). Multiple intrinsic and extrinsic factors, including genetic predisposition, chronic noise exposure, and ototoxic insults, contribute to its onset and severity (Uraguchi et al., 2021; Tarnovsky et al., 2023; Lin, 2024). Reactive oxygen species (ROS) accumulation and mitochondrial dysfunction are recognized as critical mechanisms driving cochlear degeneration during aging (He et al., 2021; Liu et al., 2023; Tan and Song, 2023). Excess D-galactose (D-gal) metabolism produces ROS, initiating a cascade of oxidative stress, inflammation, mitochondrial impairment, and apoptosis, consistent with age-associated pathology (Azman and Zakaria, 2019; Hong et al., 2023). The D-gal-induced aging model is widely established and serves as a reliable experimental paradigm to investigate aging-related biological processes and auditory pathophysiology mechanisms.

Forkhead box G1 (FOXG1) is a nuclear transcription factor indispensable for the proper development of the nervous system (Gil et al., 2024). In the auditory system, FOXG1 is integral to cochlear morphogenesis and the differentiation of hair cells (HCs), supporting cells (SCs), and spiral ganglion neurons (SGNs) (Jahan et al., 2018; Ding et al., 2020; Zhang et al., 2020). In addition, FOXG1 regulates cellular redox balance by maintaining mitochondrial integrity and controlling ROS production (He et al., 2021; Mu et al., 2023). Our previous work demonstrated that upregulation of FOXG1 expression activates autophagy, enabling HCs to resist oxidative stress-induced injury (He et al., 2021; Mu et al., 2023); however, the underlying mechanisms governing this protective interaction remains elusive. Therefore, this study sought to elucidate the role of FOXG1 in age-related cochlear HCs damage using a D-gal-induced aging model.

Among age-associated molecular alterations, autophagy dysregulation has emerged as a key hallmark of aging (Guo et al., 2022). Autophagy, a conserved catabolic process, is fundamental to maintaining cellular equilibrium through the breakdown of defective organelles and misfolded polypeptides (Klionsky et al., 2021). Under physiological and pathological states, autophagy acts as a cytoprotective mechanism that eliminates defective mitochondria and mitigates oxidative stress-induced cellular injury (Li et al., 2021; Chen et al., 2023). In the auditory system, autophagy is indispensable for normal cochlear development and the preservation of inner ear cellular functions, particularly in HCs (Yuan et al., 2018; Guo et al., 2021; He et al., 2021). Increasing evidence indicates that autophagic activity declines with age, leading to the accumulation of depolarized mitochondria and excessive ROS in HCs (Wu et al., 2020). Insufficient clearance of these impaired mitochondria can trigger apoptotic cascades and contribute to HC degeneration. Conversely, activation of the autophagy pathway promotes HC survival by sequestering damaged mitochondria into autophagosomes, which subsequently fuse with lysosomes for degradation (He et al., 2017; Li et al., 2023; Zhang et al., 2023). Although our previous studies suggested a link between FOXG1 expression and autophagy during HC degeneration in ARHL, the mechanistic interplay between these processes remains to be elucidated.

Beyond autophagy, growing evidence underscores the pivotal contribution of epigenetic alterations in the aging process. Epigenetic mechanisms orchestrate gene expression programs that regulate development, differentiation, and tissue maintenance, while their dysregulation contributes to diverse age-related diseases, including cancer (Hogg et al., 2020), neurodegeneration (Duan et al., 2022; la Torre et al., 2023) and metabolic disorders (King and Skinner, 2020). These mechanisms include DNA methylation, histone modifications, and non-coding RNA regulation, which collectively modulate chromatin accessibility and transcriptional activity (Gjaltema and Rots, 2020). Among these, histone lysine methylation, particularly histone H3 lysine 9 dimethylation (H3K9me2), is tightly connected with chromatin condensation and transcriptional repression. Aberrant H3K9me2 accumulation has been implicated in various aging-related pathologies, including neurodegenerative diseases and sensory dysfunctions (Casciello et al., 2017). For instance, elevated H3K9me2 levels have been detected in the occipital cortex of Alzheimer’s disease (AD) patients relative to age-matched healthy controls (Lithner et al., 2013). Similarly, transgenic mouse models harboring familial AD (FAD) mutations exhibit significantly increased H3K9me2 expression in the prefrontal cortex and hippocampus. This aberrant epigenetic modification contributes to the transcriptional repression of glutamate receptor genes, leading to impaired synaptic transmission and AD-like cognitive deficits. Pharmacological inhibition of H3K9me2 restores glutamate receptor expression, improves synaptic function, and alleviates cognitive impairment in aged FAD mice (Zheng et al., 2019). Likewise, in models of noise-induced hearing loss, increased H3K9me2 levels in the basal turn outer HCs (OHCs) of the cochlea have been associated with enhanced cell vulnerability, whereas inhibition of H3K9me2 mitigates OHC loss (Xiong et al., 2019). Although epigenetic regulation is increasingly recognized as essential in cochlear homeostasis and auditory pathology, the specific H3K9me2-dependent modulation of FOXG1 in ARHL remains unexplored.

In addition to histone modifications, miRNAs serve as key post-transcriptional epigenetic regulators of gene activity. These small non-coding RNAs are engaged in diverse cell processes, including growth, differentiation, apoptosis, tumorigenesis, and embryogenic development (Saliminejad et al., 2019; Huang and Choo, 2023; Maraghechi et al., 2023). Emerging evidence indicates that several miRNAs contribute to HCs maturation, SCs plasticity, and neural differentiation within the auditory system (Weston et al., 2006; Rudnicki et al., 2014; Jiang et al., 2022). Weise et al. demonstrated that FOXG1 can function as an upstream regulator of specific miRNA expression, thereby influencing downstream signaling pathways (Weise et al., 2019). Our previous study demonstrated that FOXG1 induced autophagy activation through miR-34a, miR-96, miR-182, and miR-183, thereby mediating cisplatin-induced hair cell degeneration (Mu et al., 2023). However, whether FOXG1 regulates autophagy-related miRNAs in the context of ARHL remains unexplored.

In this study, we developed a D-gal-induced senescence model using C57BL/6J mice and the inner ear HC line HEI-OC1. We analyzed the expression levels of FOXG1, autophagy-associated markers, miRNAs, and the histone modification H3K9me2 to elucidate potential mechanistic interactions among these factors. Additionally, we integrated proteomic, small RNA sequencing (RNA-seq), and metabolomic analyses in D-gal-induced aging models to comprehensively investigate the functions and regulatory roles of FOXG1 in ARHL from a multi-omics perspective.

## 2 Materials and methods

### 2.1 Animals

Male C57BL/6J mice at 5 weeks of age were sourced from SPF Biotechnology Co., Ltd. (Beijing, China). Baseline hearing was confirmed via auditory brainstem response (ABR) measurements. After a 1-week habituation phase, the mice were randomly allocated into two groups: a control group and a D-gal-induced aging group. Mice in the aging group received daily subcutaneous administrations of D-gal (200 mg/kg; #G0625; Sigma-Aldrich) over an 8-week period, while control mice received an equal volume of saline (200 mg/kg/day) under identical conditions. After completion of the injection protocol, all mice were maintained until 27 weeks of age. ABR testing was subsequently performed to evaluate hearing thresholds. Euthanasia was performed via intraperitoneal administration of pentobarbital sodium at a dose of 150 mg/kg. All animal experiments in this study were designed and reported in accordance with the ARRIVE guidelines. All procedures complied with institutional ethical standards and were approved by the Institutional Animal Care and Use Committee of Zhongnan Hospital, Wuhan University (ZN2023226).

### 2.2 Cell culture

The mouse auditory hair-cell line HEI-OC1 was propagated in Dulbecco’s modified Eagle’s medium (DMEM; Gibco) enriched with 10% fetal bovine serum (FBS; Gibco) and maintained at 33°C within a humidified incubator with 5% CO2. Upon attaining 80–90% confluence, cells were detached using 0.25% trypsin-EDTA (Gibco) for subculturing. For injury induction, HEI-OC1 cells were exposed to D-gal (30 mg/mL) for 72 h to mimic aging-associated stress.

### 2.3 ABR test

After receiving 50 mg/kg pentobarbital sodium administered intraperitoneally for anesthesia, mice were placed on a thermoregulated heating pad within a soundproof enclosure. Subdermal electrodes were positioned at the vertex (recording electrode), the test ear (reference), and the contralateral ear (ground). ABRs were acquired using a Tucker Davies Technologies (TDT, Gainesville, FL, United States) setup. Click and tone-burst stimuli were delivered at frequencies ranging from 4 to 32 kHz (4, 8, 16, 24, and 32 kHz). Stimulus intensity began at 90 dB sound pressure level (SPL) and were reduced in 10-dB increments until Wave II was no longer detectable. The hearing threshold for each frequency was determined as the minimal sound level at which a reproducible ABR waveform could be observed.

### 2.4 Immunofluorescence staining

Cochlear specimens or HEI-OC1 cells were fixed with 4% paraformaldehyde for 20 min, followed by three 5-min washes with phosphate-buffered saline (PBS). Samples were permeabilized using 2% Triton X-100 for 15 min and nonspecific binding was blocked with 10% donkey serum for 1 h at room temperature. Primary antibodies included those targeting FOXG1 (ab18259; Abcam), LC3B (L7543; Sigma-Aldrich), H3K9me2 (ab176882; Abcam), and Myosin7a (25-6790; Proteintech). Microfilaments were visualized with rhodamine-phalloidin (Yeasen), and nuclei were labeled with DAPI (Solarbio). After overnight incubation with primary antibodies at 4°C, samples were treated with appropriate fluorescent secondary antibodies and DAPI under light-protected conditions. Excess dye was removed by PBS washing, and samples were mounted using an anti-fluorescence quencher. Imaging was conducted using a confocal microscope.

### 2.5 Cell Counting Kit-8 (CCK-8)

HEI-OC1 cells were seeded into 96-well plates at the appropriate density and exposed to graded concentrations of the indicated agents. After treatment, culture medium was aspirated, and 100 μL of 10% CCK-8 reagent solution was introduced into each well. Following incubation at 37 °C for the specified duration, absorbance at 450 nm was recorded with a microplate reader to assess cellular viability.

### 2.6 Flow cytometry

Apoptosis was assessed via an Annexin V-FITC/propidium iodide (PI) double-staining assay (BD Biosciences). Following collection, cells were washed twice with PBS, and reconstituted in binding buffer. The cell suspension was stained with Annexin V-FITC and PI, gently mixed, and incubated for 15 min at room temperature under dark conditions before immediate flow cytometric analysis. Fluorescence was detected on a CytoFLEX flow cytometer (Beckman Coulter, Brea, CA, United States) equipped with a 488 nm argon laser. Annexin V-FITC was detected using a 525/40 nm bandpass filter (FL1 channel), and PI was detected using a 585/42 nm bandpass filter (FL2 channel).

To quantify mitochondrial ROS, cells were incubated with the mitochondrial superoxide probe Mito-SOX Red at a final concentration of 500 nM in PBS for 15 min at 37°C in darkness. After washing twice with pre-warmed PBS, cells were resuspended in PBS and fluorescence was immediately detected on a CytoFLEX flow cytometer (Beckman Coulter, Brea, CA, United States) using a 488 nm excitation laser and a 585/42 nm bandpass filter (FL2 channel).

### 2.7 Mitochondrial DNA (mtDNA) common deletion analysis

HEI-OC1 cells from the control and D-gal groups were collected separately. Total DNA was extracted using the TIANGEN Cell Genomic DNA Extraction Kit according to the manufacturer’s instructions. The mtDNA common deletion ratio was measured by Taqman PCR. The primers and probes used for TaqMan PCR detection of the D-loop region were as follows: Forward: 5’-GGT TCT TAC TTC AGG GCC ATC A; Reverse: 5’-GAT TAG ACC CGT TAC CAT CGA GAT; probe: 5’-FAM-TTG GTT CAT CGT TAC GTT CCC CTT A-TAMRA. The primers and probes used for TaqMan PCR detection of the common deletion were as follows: Forward: 5’-AAG GAC GAA CCT GAG CCC TAA TA; Reverse: 5’- CGA AGT AGA TGA TCC GTA TGC TGT A; probe: 5’-FAM- TCA CTT TAA TCG CCA CAT CCA TAA CTG CTG T-TAMRA.

### 2.8 Transmission electron microscopy (TEM)

HEI-OC1 cells were harvested and fixed in 2.5% glutaraldehyde for 24 h, followed by fixation with 1% osmium tetroxide for 1–2 h. Specimens underwent dehydration through a graded ethanol series before being embedded in Araldite CY 212 epoxy resin (TAAB). Ultrathin sections were cut, sequentially stained with uranyl acetate and alkaline lead citrate, rinsed with distilled water, and visualized using a transmission electron microscope (JEM-1230).

### 2.9 Transfection

Small interfering RNA (siRNAs) targeting Foxg1 and Foxg1 overexpression plasmid were custom-synthesized by GenePharma Co. (Shanghai, China). MiRNA inhibitors and mimics were acquired from RiboBio Co., Ltd. (Guangzhou, China).

HEI-OC1 cells were plated in 6-well plates and grown to approximately 50–60% confluence before transfection. Transfections were performed using Lipofectamine 3000 (Thermo Fisher Scientific) following the supplier’s instructions. In brief, siRNA (or plasmid DNA), miRNA mimics, or inhibitors were diluted in Opti-MEM, combined with the transfection reagent, and allowed to incubate for 15 min at room temperature. The resulting complexes were gently added to the cells in a dropwise manner. After 6–8 h, the transfection mixture was replaced with fresh complete medium, and cells were cultured for an additional 24 h before subsequent treatments.

### 2.10 Western blot analysis

Proteins were extracted by lysing cells in pre-chilled radioimmunoprecipitation assay (RIPA) buffer containing phenylmethylsulfonyl fluoride (PMSF), protease inhibitor cocktails II and III, and phosphatase inhibitors (Roche, Basel, Switzerland). Tissue samples from mouse cochleae were homogenized in the same buffer using a mechanical grinder, followed by incubation on ice for 30 min. Lysates were centrifuged at 12,000 × g for 15 min at 4 °C to obtain the supernatants. Protein concentrations were quantified using a bicinchoninic acid (BCA) assay kit (Thermo Fisher Scientific).

Equivalent protein amounts were resolved by SDS-PAGE and electroblotted onto PVDF membranes (Millipore). Membranes were blocked in 5% nonfat milk in TBST for 1 h at room temperature and subsequently incubated overnight at 4°C with the following primary antibodies: anti-H3K9me2 (ab176882; Abcam), anti-FOXG1 (ab18259; Abcam), anti-LC3B (L7543; Sigma-Aldrich), anti-G9a (ab185050; Abcam), and anti-GAPDH antibody (10494-1-AP; Proteintech). Following three washes with TBST, membranes were probed with HRP-conjugated secondary antibodies (Antgene; ANT020) diluted 1:5,000 for 1 h at room temperature. Band intensity was detected with an enhanced chemiluminescence (ECL) kit (E423; Vazyme), and analyzed with ImageJ software.

### 2.11 Quantitative real-time PCR (qRT-PCR) analysis

Total RNA was isolated from HEI-OC1 cells and mouse cochlear tissues with the TRIzol reagent (R411-01; Vazyme) following the supplier’s instructions. Complementary DNA (cDNA) synthesis was carried out using a reverse transcription kit (Takara). qRT-PCR was conducted with the Takara qPCR Master Mix on a real-time PCR detection instrument. GAPDH served as the endogenous reference gene for normalization of target gene expression. All the primer sequences are provided in Supplementary Table S1. Primers sets for miRNAs were purchased from RiboBio Co. (Guangzhou, China).

### 2.12 Proteomics analysis

HEI-OC1 cells (control, D-gal, and si-FOXG1 groups) and mouse cochlear (control and aging groups) were collected for proteomic profiling (Novogene, Beijing, China), with three independent biological replicates per condition. Differentially expressed proteins (DEPs) were defined as those with a *p*-value < 0.05 and | log2 fold change (FC)| > 1.2. Functional enrichment analyses, including Gene Ontology (GO) and Kyoto Encyclopedia of Genes and Genomes (KEGG) pathway assessments, were executed using the ClusterProfiler package in R to evaluate the biological roles and signaling networks linked to the identified DEPs.

### 2.13 Small RNA-seq

HEI-OC1 cells from control, D-gal, and si-FOXG1 groups were collected for small RNA sequencing (Novogene, Beijing, China), with triplicate biological samples per group. Total RNA was isolated using the TRIzol, and libraries were prepared and sequenced in accordance with Illumina standard workflows. Differentially expressed miRNAs (DEmiRNAs) were identified via the DESeq2 package in R, applying thresholds of p < 0.05 and | log2 FC| > 1.2. Potential miRNA targets were predicted using both miRanda and RNAhybrid algorithms, and intersecting targets were selected for downstream analysis. Functional enrichment of target genes was conducted through GO and KEGG pathway assessments with the clusterProfiler package in R, applying a significance cut-off of *p* < 0.05.

### 2.14 Metabolomics analysis

HEI-OC1 cells from the control, D-gal, and D-gal combined with si-FOXG1 groups were collected for untargeted metabolomic profiling (Novogene, Beijing, China), with three independent replicates per group. Metabolite annotation was performed using the KEGG, the Human Metabolome Database (HMDB), and the LIPID Metabolites and Pathways Strategy (LIPIDMaps) databases. Differential metabolites were identified based on the following criteria: variable importance in projection (VIP) > 1, *p* < 0.05, and FC > 1.2. K-means clustering (*k* = 4) was performed on the differential metabolites.

### 2.15 Statistical analysis

All data are expressed as mean ± standard deviation (SD). Statistical evaluations were conducted with Microsoft Excel and GraphPad Prism 8. Intergroup comparisons involving two conditions were analyzed using two-tailed unpaired Student’s *t*-tests, whereas comparisons across three or more groups were assessed by one-way ANOVA with Dunnett’s multiple comparison test. Statistical significance was defined as *p* < 0.05.

## 3 Results

### 3.1 Construction of mimetic aging mouse and cell models with D-gal

**FIGURE 1:**
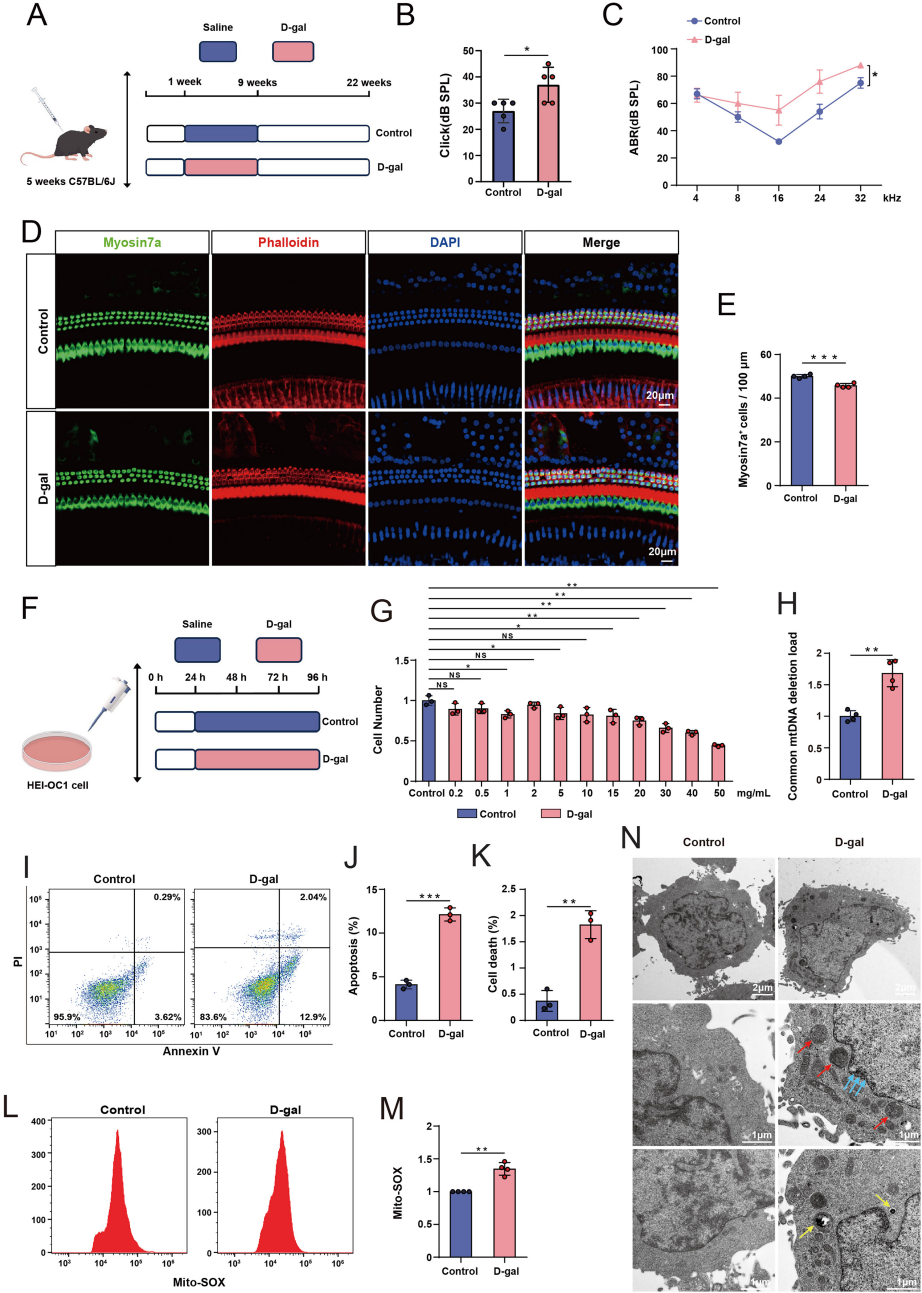
Construction of mimetic aging mouse and cell models with D-gal. **(A)** Experimental protocol for D-gal-induced mimetic aging mice. **(B)** Statistical analysis of the click ABR between the control group and the D-gal group (*n* = 5). **(C)** Statistical analysis of tone burst ABR in different frequencies between the control group and the D-gal group (*n* = 5). **(D)** Immunofluorescence staining of the cochlear from the control mice and the D-gal mice with Myosin7a (green), Phalloidin (red) and DAPI (blue). **(E)** Quantification of OHCs in the control group and the D-gal group (*n* = 4). **(F)** Experimental protocol for D-gal-induced mimetic aging cells. **(G)** CCK-8 assay results for HEI-OC1 cells after 72-h treatment with different concentrations of D-gal (*n* = 3). **(H)** Quantification of the mtDNA CD levels in the control group and the D-gal group (*n* = 4). **(I)** Flow cytometric analysis of apoptosis in the control group and the D-gal group. **(J)** Quantification of apoptotic cells in the control group and the D-gal group (*n* = 3). **(K)** Quantification of dead cells in the control group and the D-gal group (*n* = 3). **(L)** Flow cytometric analysis of ROS in the control group and the D-gal group. **(M)** Quantification of ROS levels in the control group and the D-gal group (*n* = 4). **(N)** TEM results of structural changes in cells from the control group and the D-gal group (Red arrows: mitochondrial pathology, Blue arrows: chromatin condensation, Yellow arrows: cytoplasmic accumulation). Control cells received an equal volume of saline. NS, not significant, *p < 0.05, **p < 0.01, ***p < 0.001.

The D-gal-induced aging model is widely used to recapitulate phenotypes characteristic of natural aging. Following established protocols (He et al., 2021; Wang et al., 2023; Chen F. et al., 2024), C57BL/6J mice received daily subcutaneous D-gal injection (200 mg/kg) for 8 weeks; control mice received matched saline (Figure 1A). ABR showed a significant elevation of click thresholds in D-gal-treated mice (Figure 1B) and a significant threshold shift at 32 kHz on tone-burst ABR (Figure 1C). Low-frequency changes trended upward but were not significant (Figure 1C). These findings align with the reports of hearing loss in naturally aging mice. Consistent with functional deficits, immunofluorescence for Myosin7a/Phalloidin/DAPI revealed a significant loss of OHCs in D-gal-treated cochleae (Figures 1D,E).

HEI-OC1 cells, which express multiple inner-ear HC markers, were exposed to D-gal (0.2–50 mg/mL) for 72 h to establish a cell-based aging model (Figure 1F). The CCK-8 assays demonstrated a concentration-dependent loss of viability, with a significant reduction at 30 mg/mL (Figure 1G); this dose and duration were used for subsequent senescence phenotyping. D-gal increased the mtDNA common deletion load (Figure 1H), elevated apoptosis and cell death by Annexin V/PI flow cytometry (Figures 1I–K), and raised mitochondrial ROS by Mito-SOX flow cytometry (Figures 1L,M). TEM revealed canonical senescence-associated ultrastructural changes, including nuclear degeneration, chromatin condensation, cytoplasmic accumulation, and mitochondrial pathology (Figure 1N). Collectively, the D-gal models reproduced key functional and structural features of natural aging.

### 3.2 The levels of FOXG1, H3K9me2, and autophagy are changed after D-gal treatment

**FIGURE 2:**
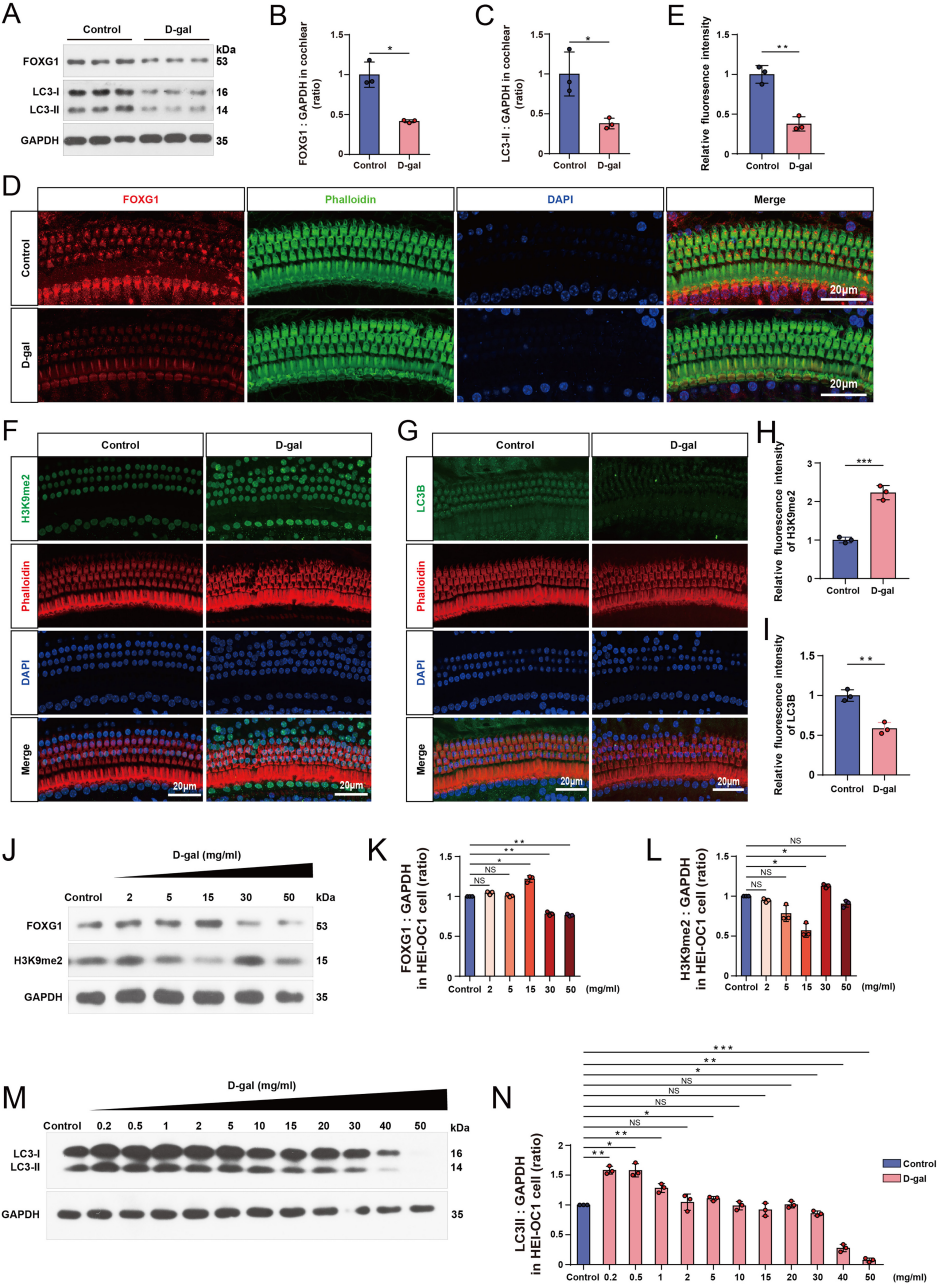
Changes in FOXG1, H3K9me2, and autophagy levels following D-gal treatment. **(A)** Western blot of FOXG1 and LC3 expression levels in the cochlear of the control mice and the D-gal mice. D-gal (200 mg/kg/day) was administered via subcutaneous injection for 8 weeks. **(B)** Quantitative analysis of the FOXG1 levels in **(A)** (*n* = 3). **(C)** Quantitative analysis of the LC3-II levels in **(A)** (*n* = 3). **(D)** Immunofluorescence staining of the cochlear from the control mice and the D-gal mice with anti-FOXG1 (red), Phalloidin (green) and DAPI (blue). **(E)** Quantification of FOXG1 expression in **(D)** (*n* = 3). **(F)** Immunofluorescence staining of the cochlear from the control mice and the D-gal mice with anti-H3K9me2 (green), Phalloidin (red) and DAPI (blue). **(G)** Immunofluorescence staining of the cochlear from the control mice and the D-gal mice with anti-LC3B (green), Phalloidin (red) and DAPI (blue). **(H)** Quantification of H3K9me2 expression in **(F)** (*n* = 3). **(I)** Quantification of LC3B expression in **(G)** (*n* = 3). **(J)** Western blot of FOXG1 and H3K9me2 expression levels in the HEI-OC1 cells after 72-h treatment with different concentrations of D-gal. **(K)** Quantitative analysis of the FOXG1 levels in **(J)** (*n* = 3). **(L)** Quantitative analysis of the H3K9me2 levels in **(J)** (*n* = 3). **(M)** Western blot of LC3 expression levels in the HEI-OC1 cells after 72-h treatment with different concentrations of D-gal. **(N)** Quantitative analysis of the LC3-II levels in **(M)** (*n* = 3). NS, not significant, *p < 0.05, **p < 0.01, ***p < 0.001.

To examine the molecular alterations induced by D-gal in the cochlea, we analyzed the expression of FOXG1, H3K9me2, and autophagy-related proteins. Western blotting revealed that FOXG1 and LC3 protein levels were significantly reduced in the cochlea of D-gal-treated mice compared with controls (Figures 2A–C). Immunofluorescence confirmed the downregulation of FOXG1 (Figures 2D,E) and LC3 (Figures 2G,I), consistent with western blot results. Because epigenetic dysregulation contributes to age-related oxidative and neurodegeneration processes, we further examined histone methylation changes in the cochlea. H3K9me2, a key epigenetic mark involved in chromatin silencing, transcriptional regulation, and redox-responsive cellular adaptation (Yu et al., 2013; Tang et al., 2016; Xiong et al., 2019), plays an essential role in maintaining euchromatin structure and regulating gene repression. Consistent with its reported role in neurodegenerative diseases, H3K9me2 expression was markedly elevated in the cochleae of D-gal-treated compared with controls (Figures 2F,H). Next, we treated HEI-OC1 cells with graded concentrations of D-gal and examined FOXG1, H3K9me2, and LC3 expression by western blotting. FOXG1 expression increased at 2–15 mg/mL but declined significantly when the D-gal concentration exceeded 15 mg/mL. In contrast, H3K9me2 levels exhibited the opposite trend (Figures 2J–L). LC3 expression paralleled the pattern of FOXG1 (Figures 2M,N), suggesting that autophagic activity followed FOXG1 regulation under redox imbalance. These data collectively indicate that FOXG1, H3K9me2, and autophagy respond dynamically to D-gal-induced aging.

### 3.3 Elevated FOXG1 levels and autophagy activation after inhibition of H3K9me2 by BIX01294

**FIGURE 3:**
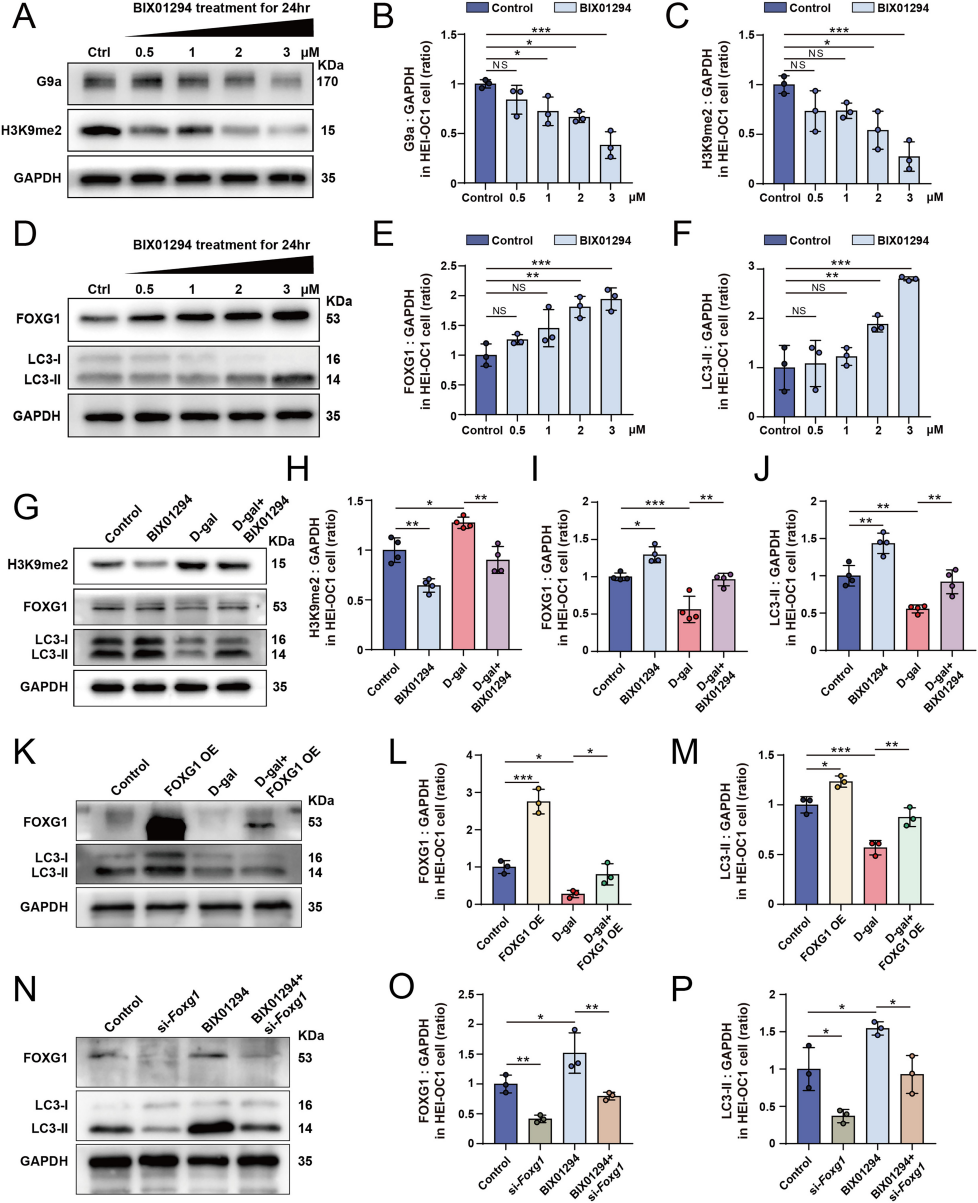
H3K9me2 may regulate autophagy through FOXG1. **(A)** Western blot of G9a and H3K9me2 expression levels in the HEI-OC1 cells after 24-h treatment with different concentrations of BIX01294. **(B)** Quantitative analysis of the G9a levels in **(A)** (*n* = 3). **(C)** Quantitative analysis of the H3K9me2 levels in **(A)** (*n* = 3). **(D)** Western blot of FOXG1 and LC3 expression levels in the HEI-OC1 cells after 24-h treatment with different concentrations of BIX01294. **(E)** Quantitative analysis of the FOXG1 levels in **(D)** (*n* = 3). **(F)** Quantitative analysis of the LC3-II levels in **(D)** (*n* = 3). **(G)** Western blot of H3K9me2, FOXG1 and LC3 expression levels in the HEI-OC1 cells following D-gal and BIX01294 treatment. **(H)** Quantitative analysis of the H3K9me2 levels in G (*n* = 4). **(I)** Quantitative analysis of the FOXG1 levels in **(G)** (*n* = 4). **(J)** Quantitative analysis of the LC3-II levels in **(G)** (*n* = 4). **(K)** Western blot of FOXG1 and LC3 expression levels in the HEI-OC1 cells following D-gal treatment and FOXG1 overexpression. **(L)** Quantitative analysis of the FOXG1 levels in **(K)** (*n* = 3). **(M)** Quantitative analysis of the LC3-II levels in **(K)** (*n* = 3). **(N)** Western blot of FOXG1 and LC3 expression levels in the HEI-OC1 cells following BIX01294 treatment and FOXG1 knockdown. **(O)** Quantitative analysis of the FOXG1 levels in **(N)** (*n* = 3). **(P)** Quantitative analysis of the LC3-II levels in **(N)** (*n* = 3). NS, not significant, *p < 0.05, **p < 0.01, ***p < 0.001.

G9a (euchromatic histone-lysine N-methyltransferase 2; EHMT2) and GLP (G9a-like protein; EHMT1) are the primary functional methyltransferases responsible for H3K9me2 methylation. BIX01294, a selective inhibitor of these enzymes, is commonly used to investigate the biological functions of H3K9me2. HEI-OC1 cells were treated with increasing concentrations of BIX01294 (0.5, 1, 2, 3, and 5 μM) for 24 h. CCK-8 assays revealed a dose-dependent decrease in cell viability following BIX01294 treatment (Supplementary Figure S1A). Consistently, flow cytometry demonstrated a concentration-dependent increase in apoptosis and cell death ratios (Supplementary Figures S1B–D). Western blotting showed that the expression of G9a and H3K9me2 decreased progressively with increasing BIX01294 concentrations (Figures 3A–C). To determine whether H3K9me2 affects FOXG1 and autophagy, we analyzed FOXG1 and LC3 expression in BIX01294-treated HEI-OC1 cells. The results revealed a dose-dependent upregulation of both FOXG1 and LC3, suggesting that suppression of H3K9me2 enhances FOXG1 expression and autophagic activity (Figures 3D–F). Collectively, these findings indicate that pharmacological inhibition of G9a by BIX01294 effectively reduces H3K9me2 levels and concomitantly increases FOXG1 and LC3 expression, implying that H3K9me2 exerts an inhibitory effect on FOXG1 and autophagy in HEI-OC1 cells. Subsequent experiments were performed using 2 μM BIX01294 for 24 h.

### 3.4 H3K9me2 may regulate autophagy through FOXG1

To elucidate whether H3K9me2 is associated with FOXG1 and autophagy, we pretreated HEI-OC1 cells with BIX01294 for 24 h prior to D-gal exposure and found that BIX01294 attenuated the D-gal-induced reduction in both FOXG1 and LC3 expression levels (Figures 3G–J). We then silenced FOXG1 expression in HEI-OC1 cells using siRNA and examined the levels of FOXG1 and LC3 by western blotting. The results showed that the levels of LC3-II were significantly decreased following FOXG1 knockdown (Supplementary Figures S2A–C). Next, we constructed a FOXG1-overexpression plasmid. Following transfection, western blotting confirmed markedly elevated FOXG1 expression, accompanied by a concomitant increase in LC3-II levels (Supplementary Figures S2D–F). We also found that FOXG1 overexpression mitigated the D-gal-induced suppression of autophagy in HEI-OC1 cells (Figures 3K–M). These findings suggest that FOXG1 positively regulates autophagy during D-gal-induced oxidative injury. To further explore whether H3K9me2 regulates autophagy through FOXG1, we knocked down FOXG1 and treated the cells with BIX01294. Western blotting showed that autophagy was enhanced by BIX01294 treatment alone but markedly reduced when silenced prior to BIX01294 exposure (Figures 3N–P). These results indicate that H3K9me2 regulates autophagy via a FOXG1-dependent mechanism, and FOXG1 knockdown abolishes the BIX01294-induced activation of autophagy.

### 3.5 FOXG1-induced autophagy activation may be mediated by autophagy-related miRNAs

**FIGURE 4:**
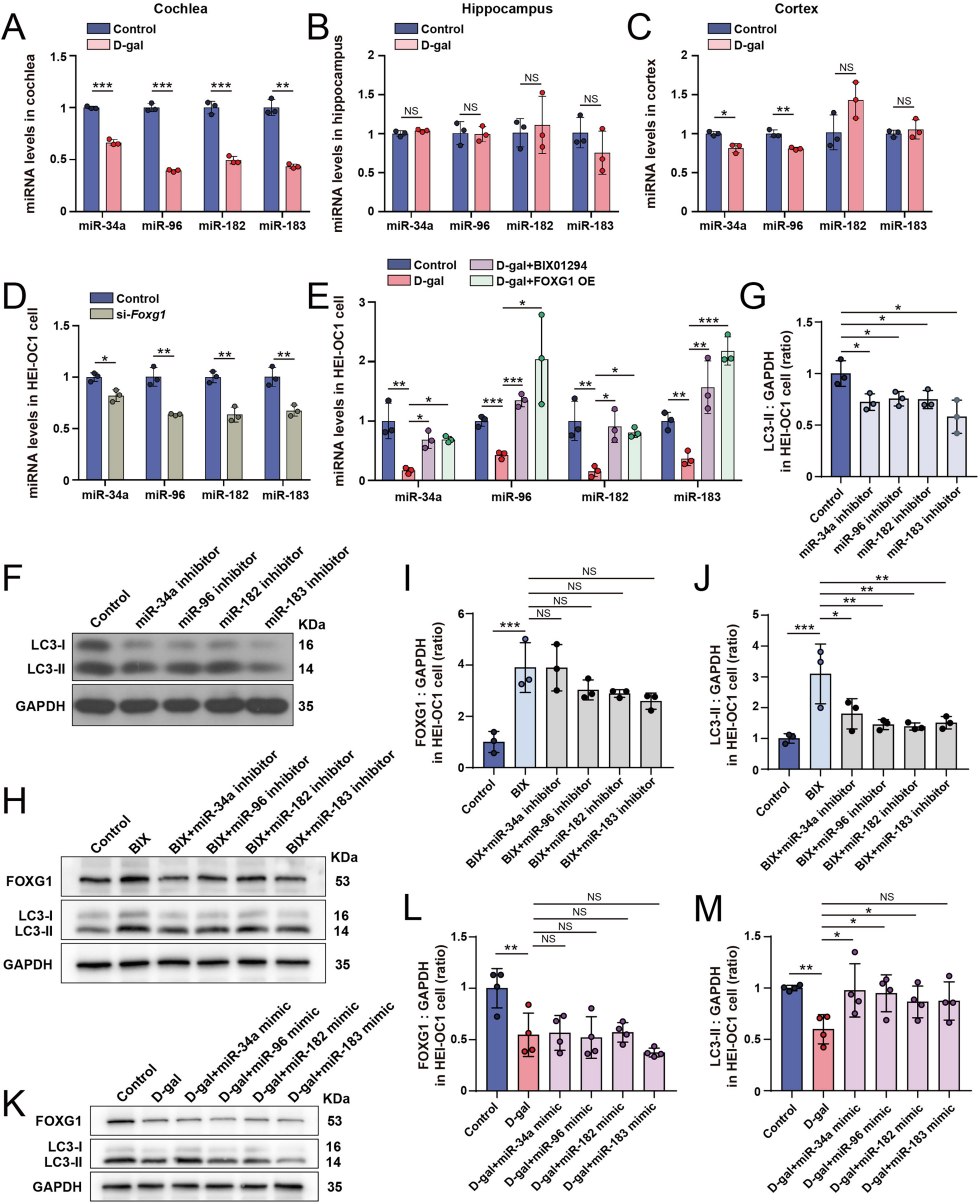
FOXG1-induced autophagy activation may be mediated by autophagy-related miRNAs. **(A)** Expression levels of miR-34a, miR-96, miR-182, and miR-183 in the cochlea of the control mice and the D-gal mice (*n* = 3). **(B)** Expression levels of miR-34a, miR-96, miR-182, and miR-183 in the hippocampus of the control mice and the D-gal mice (*n* = 3). **(C)** Expression levels of miR-34a, miR-96, miR-182, and miR-183 in the cortex of the control mice and the D-gal mice (*n* = 3). **(D)** Expression levels of miR-34a, miR-96, miR-182, and miR-183 in the HEI-OC1 cells after knocking down FOXG1 (*n* = 3). **(E)** Expression levels of miR-34a, miR-96, miR-182, and miR-183 in HEI-OC1 cells treated with D-gal after BIX01294 pretreatment or FOXG1 overexpression (*n* = 3). **(F)** Western blot of LC3 expression levels in the HEI-OC1 cells following inhibition of miR-34a, miR-96, miR-182, and miR-183. **(G)** Quantitative analysis of the LC3-II levels in **(F)** (*n* = 3). **(H)** Western blot of FOXG1 and LC3 expression levels in the HEI-OC1 cells treated with BIX01294 combined with miR-34a, miR-96, miR-182, and miR-183 inhibitors, respectively. **(I)** Quantitative analysis of the FOXG1 levels in **(H)** (*n* = 3). **(J)** Quantitative analysis of the LC3-II levels in **(H)** (*n* = 3). **(K)** Western blot of FOXG1 and LC3 expression levels in the HEI-OC1 cells treated with D-gal combined with miR-34a, miR-96, miR-182, and miR-183 mimics, respectively. **(L)** Quantitative analysis of the FOXG1 levels in **(K)** (*n* = 4). **(M)** Quantitative analysis of the LC3-II levels in K (*n* = 4). NS, not significant, *p < 0.05, **p < 0.01, ***p < 0.001.

While our previous studies suggested a connection between FOXG1 and autophagy, the precise molecular mechanism driving this relationship remains to be fully elucidated. MiRNAs are known to regulate different stages of autophagy, and their expression is regulated by transcription factors. We hypothesized that miRNAs act as potential mediators between FOXG1 expression and autophagy. To verify this hypothesis and based on previous publications (Mu et al., 2023), we examined the expression of miRNAs that not only correlate with autophagy but also function in the inner ear, and analyzed their expression in D-gal-induced aging models, including miR-34a, miR-96, miR-182, and miR-183. We extracted RNA from the cochlea, hippocampus, and cortex of D-gal-induced aging mice and detected the expression of these miRNAs via qRT-PCR. Compared with the control group, qRT-PCR analysis revealed a significant downregulation of miR-34a, miR-96, miR-182, and miR-183 in the cochlea of D-gal-induced aging mice (Figure 4A), whereas their expression remained largely unaltered in the hippocampus and cortex (Figures 4B,C). We then silenced FOXG1 expression in HEI-OC1 cells via siRNA transfection and observed a significant reduction in the expression of miR-34a, miR-96, miR-182, and miR-183 (Figure 4D). These results suggest that these autophagy-related miRNAs are positively regulated by FOXG1. We next treated HEI-OC1 cells, pretreated with either 2 μM BIX01294 or FOXG1-overexpression plasmids, with D-gal. The qRT-PCR results showed that D-gal treatment downregulated the expression of miR-34a, miR-96, miR-182, and miR-183; however, pretreatment with BIX01294 or FOXG1-overexpression reversed the D-gal-induced decrease in miRNA expression (Figure 4E). To further investigate the functional role of these miRNAs in regulating autophagy, we inhibited their expression in HEI-OC1 cells using miRNA inhibitors (Supplementary Figure S3A) and measured LC3 expression. Following miRNA inhibition, a significant decline in LC3-II expression was observed (Figures 4F,G), indicating a reduction in autophagic activity.

**FIGURE 5:**
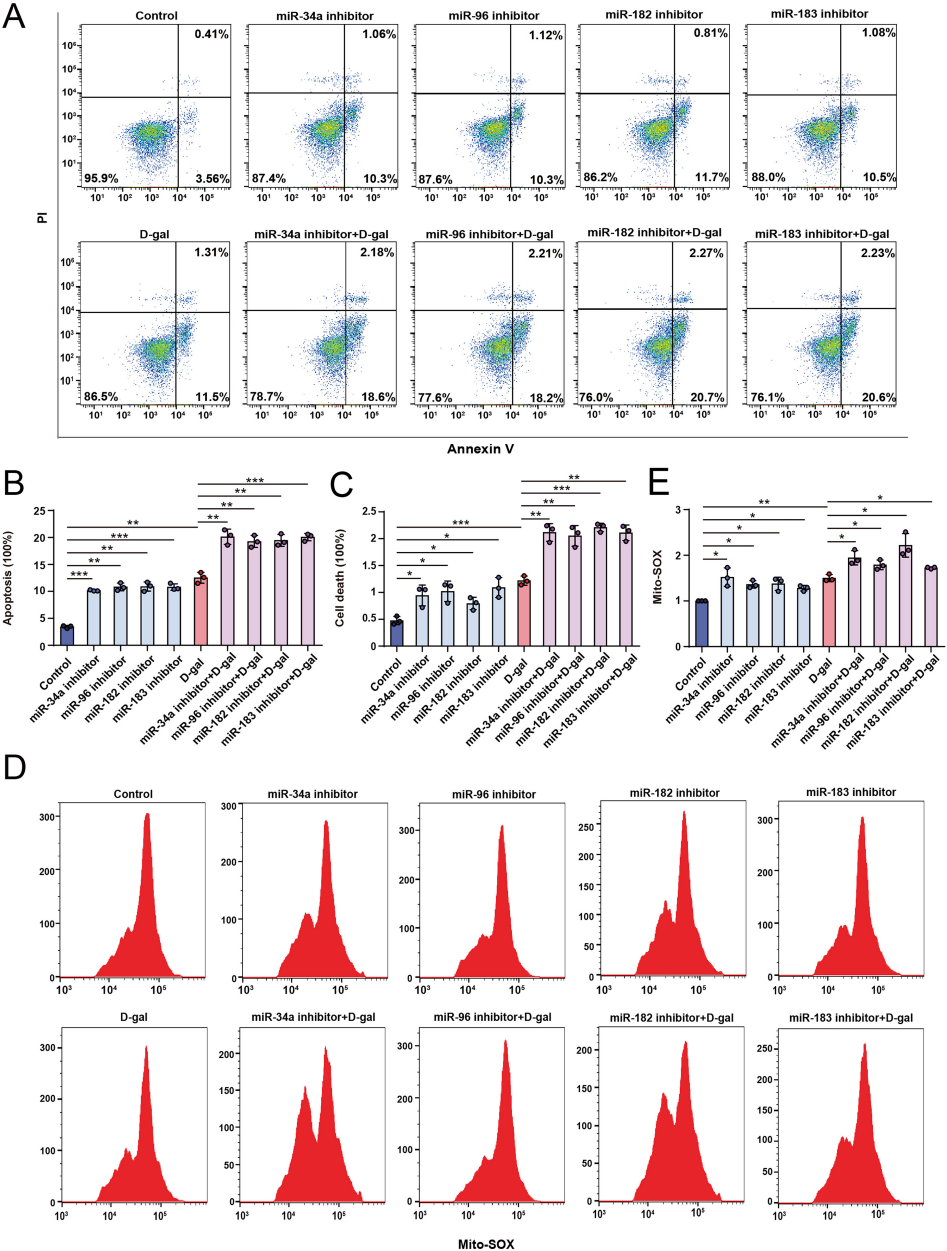
Flow cytometric analysis of apoptosis and ROS levels in the HEI-OC1 cells following inhibition of miRNAs and D-gal treatment. **(A)** Flow cytometric analysis of apoptosis in the HEI-OC1 cells following inhibition of miR-34a, miR-96, miR-182, and miR-183, and combined with D-gal treatment. **(B)** Quantification of apoptotic cells in **(A)** (*n* = 3). **(C)** Quantification of dead cells in **(A)** (*n* = 3). **(D)** Flow cytometric analysis of ROS levels in the HEI-OC1 cells following inhibition of miR-34a, miR-96, miR-182, and miR-183, and combined with D-gal treatment. **(E)** Quantification of ROS levels in **(D)** (*n* = 3). *p < 0.05, **p < 0.01, ***p < 0.001.

Next, after inhibiting the four miRNAs in HEI-OC1 cells, we treated the cells with 2 μM BIX01294 and examined FOXG1 and LC3 protein levels. Western blot analysis demonstrated that relative to BIX01294 treatment alone, co-treatment with BIX01294 and miRNA inhibitors resulted in a significant reduction in LC3-II, whereas FOXG1 levels remained unchanged (Figures 4H–J). This suggests that inhibition of these miRNAs suppresses autophagy under BIX01294 treatment, while FOXG1 expression is unaffected by miRNA inhibition. We next overexpressed miR-34a, miR-96, miR-182, and miR-183 in HEI-OC1 cells by transfection with miRNA mimics (Supplementary Figure S3B), followed by D-gal treatment, and examined FOXG1 and LC3 expression. Western blot analysis revealed that, relative to the D-gal group, LC3-II levels were increased in the D-gal + miRNA mimics group, whereas FOXG1 expression remained unaffected by miRNA mimics (Figures 4K–M). These results indicate that overexpression of these four miRNAs restores autophagy under D-gal-induced stress. Subsequently, we used flow cytometry to quantify apoptosis and cell death in HEI-OC1 cells following inhibition of these miRNAs, both with and without D-gal treatment. The proportion of apoptotic cells and cell death increased after inhibition alone and was further elevated under combined miRNA inhibition and D-gal exposure (Figures 5A–C). Consistently, Mito-SOX flow cytometry revealed a similar increase in mitochondrial ROS levels (Figures 5D,E). These observations underscore a critical protective function of these miRNAs in preserving HEI-OC1 cell viability and mitochondrial homeostasis.

In summary, these findings demonstrate an association between miR-34a, miR-96, miR-182, miR-183 and autophagic processes, and FOXG1-mediated autophagy activation may be mediated by these miRNAs.

### 3.6 Proteomics analysis of the aging mouse cochlear and the D-gal-induced aging cells

**FIGURE 6:**
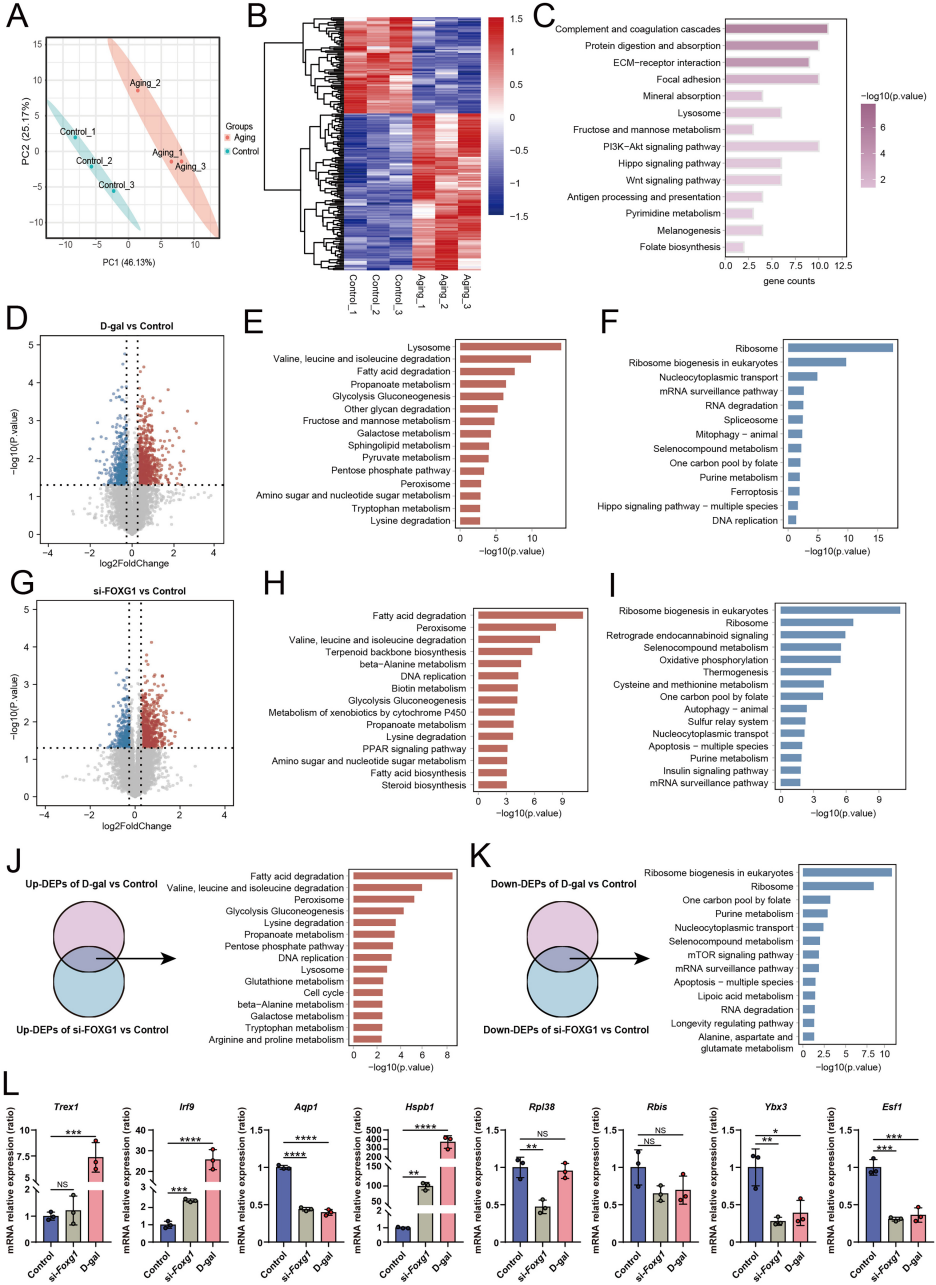
Proteomics analysis of the aging mouse cochlear and the D-gal-induced aging cells. **(A)** PCA of proteomics data from the cochlear of control and aging mice. **(B)** Heatmap of DEPs between the control group and the aging group. **(C)** KEGG enrichment analysis of DEPs between the control group and the aging group. **(D)** Volcano plot of DEPs between the control group and the D-gal group in HEI-OC1 cells (Up-DEPs shown as red dots; down-DEPs shown as blue dots). **(E)** KEGG enrichment of upregulated DEPs in the D-gal group. **(F)** KEGG enrichment of downregulated DEPs in the D-gal group. **(G)** Volcano plot of DEPs between the control group and the si-FOXG1 group in HEI-OC1 cells (Up-DEPs shown as red dots; down-DEPs shown as blue dots). **(H)** KEGG enrichment of upregulated DEPs in the si-FOXG1 group. **(I)** KEGG enrichment of downregulated DEPs in the si-FOXG1 group. **(J)** KEGG enrichment analysis of the overlapping upregulated DEPs in both the D-gal group and the si-FOXG1 group. **(K)** KEGG enrichment analysis of the overlapping downregulated DEPs in both the D-gal group and the si-FOXG1 group. **(L)** Validation results for the top four overlapping upregulated (left four bars) and downregulated (right four bars) DEPs identified in both the D-gal group and the si-FOXG1 group by qRT-PCR (*n* = 3). NS, not significant, *p < 0.05, **p < 0.01, ***p < 0.001, ****p < 0.0001.

Aging is a complex process characterized by progressive molecular and cellular changes. Given that ARHL cannot be attributed to alterations in a few genes, we employed proteomics to comprehensively analyze protein expression profiles in the aging mouse cochlea and D-gal-induced aging cells, aiming to obtain a more holistic understanding of protein alterations and associated signaling pathways involved in aging. We conducted tandem mass tag (TMT) proteomic analysis of the cochleae from 12-month-old (aging) and 8-week-old (young control) mice. To evaluate inter-group separation and data quality before differential analysis, we performed principal component analysis (PCA), which clearly distinguished the aged and young control groups (Figure 6A). Comparative analysis revealed 192 DEPs between the two groups. The expression distribution of these DEPs was visualized in the heatmap and volcano plots in Figure 6B and Supplementary Figure S4A, respectively. Specifically, 119 proteins were upregulated and 73 were downregulated in the cochleae of aging mice. GO and KEGG enrichment analyses were performed to explore the biological implications of these DEPs. GO analysis, focusing on biological processes (BP), showed that DEPs were predominantly implicated in tissue remodeling, antigen processing and presentation, fibrinolysis, vascular processes in the circulatory system, bone remodeling, and regulation of coagulation (Supplementary Figure S4B). KEGG pathway analysis indicated significant enrichment in the complement and coagulation cascades, protein digestion and absorption, extracellular matrix (ECM)-receptor interaction, lysosomes, fructose and mannose metabolism, PI3K-Akt, Hippo, and Wnt signaling pathways, alongside pyrimidine metabolism, and folate biosynthesis (Figure 6C).

We next employed rapid data-independent acquisition (DIA) proteomics to identify DEPs in HEI-OC1 cells between the D-gal and control groups and between the si-FOXG1 and control groups. The PCA results showed distinct clustering between the control and D-gal groups (Supplementary Figure S4C). Differential analysis identified 1,215 DEPs between these two conditions, of which 746 proteins were upregulated and 469 were downregulated in the D-gal group (Figure 6D). GO enrichment analysis categorized these DEPs by BP, cellular components (CC), and molecular functions (MF) (Supplementary Figure S4D). KEGG pathway analysis revealed that upregulated DEPs were notably enriched in lysosomes, peroxisomes, sphingolipid metabolism, glycolysis/ gluconeogenesis, amino sugar and nucleotide sugar metabolism, branched-chain amino acid degradation (valine, leucine, isoleucine), fatty acid degradation, and lysine degradation (Figure 6E). Conversely, the downregulated DEPs were significantly enriched in ribosome biogenesis, mitophagy, DNA replication, mRNA surveillance pathway, purine metabolism, ferroptosis, and the Hippo signaling pathway (Figure 6F). We then compared DEPs between the si-FOXG1 and control groups. PCA and volcano plots demonstrated clear separation and distribution of DEPs between the two groups (Figure 6G and Supplementary Figure S4E). A total of 941 DEPs were identified, comprising 649 upregulated and 292 downregulated proteins in the si-FOXG1 group. GO analysis revealed that these DEPs were enriched in multiple categories, including BP, MF, and CC (Supplementary Figure S4F). KEGG analysis indicated that upregulated DEPs were primarily associated with peroxisomes, glycolysis/gluconeogenesis, amino sugar and nucleotide sugar metabolism, branched-chain amino acid degradation, fatty acid degradation, and lysine degradation (Figure 6H), whereas downregulated DEPs were predominantly enriched in ribosome biogenesis, oxidative phosphorylation, autophagy, insulin signaling, mRNA surveillance, and purine metabolism (Figure 6I).

Next, we performed an overlap analysis of upregulated and downregulated DEPs in the D-gal and si-FOXG1 groups, followed by KEGG enrichment of the intersecting proteins. The overlapping upregulated DEPs were primarily enriched in peroxisomes, fatty acid degradation, branched-chain amino acid degradation, glycolysis/gluconeogenesis, lysine degradation, pentose phosphate pathway, DNA replication, lysosome, glutathione metabolism, and galactose metabolism (Figure 6J and Supplementary Table S2). These results suggest that both D-gal treatment and FOXG1 knockdown induce oxidative stress, metabolic reprogramming, and inflammatory activation in HEI-OC1 cells. The overlapping downregulated DEPs were enriched in ribosome biogenesis in eukaryotes, one-carbon pool by folate, purine metabolism, nucleocytoplasmic transport, mTOR signaling pathway, mRNA surveillance pathway, and the longevity-regulating pathway (Figure 6K and Supplementary Table S2). This pattern implies that either D-gal exposure or FOXG1 loss suppresses global protein synthesis, reduces genomic stability, and impairs anabolic metabolism in HEI-OC1 cells. Finally, Figure 6L presented qRT-PCR validation of the top four overlapping upregulated and downregulated proteins identified in both the D-gal and si-FOXG1 groups, confirming the reliability of the proteomic data.

### 3.7 Small RNA-seq analysis of the D-gal-induced aging cells

**FIGURE 7:**
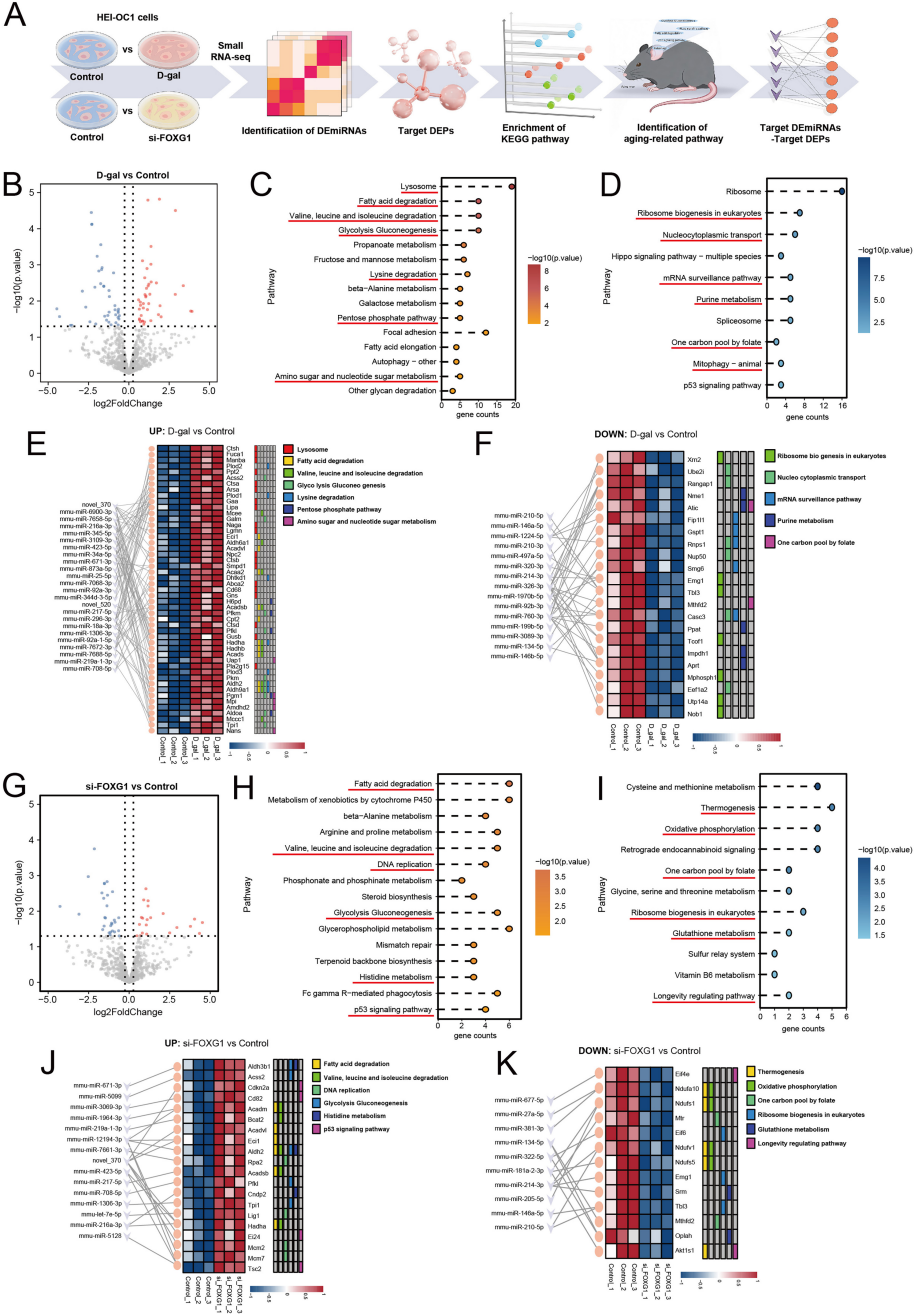
Small RNA-seq analysis of D-gal-induced aging cells and the FOXG1-knockdown cells. **(A)** Flowchart of analysis steps for small RNA-seq results. **(B)** Volcano plot of DEmiRNAs between the control group and the D-gal group (Up-DEmiRNAs shown as red dots; down-DEmiRNAs shown as blue dots). **(C)** KEGG enrichment analysis of DEPs targeted by the upregulated DEmiRNAs in the D-gal group. **(D)** KEGG enrichment analysis of DEPs targeted by the downregulated DEmiRNAs in the D-gal group. **(E)** Interactions between DEmiRNAs and their target DEPs in the upregulated aging-related pathways in **(C)**. **(F)** Interactions between DEmiRNAs and their target DEPs in the downregulated aging-related pathways in **(D)**. **(G)** Volcano plot of DEmiRNAs between the control group and the si-FOXG1 group (Up-DEmiRNAs shown as red dots; down-DEmiRNAs shown as blue dots). **(H)** KEGG enrichment analysis of DEPs targeted by the upregulated DEmiRNAs in the si-FOXG1 group. **(I)** KEGG enrichment analysis of DEPs targeted by the downregulated DEmiRNAs in the si-FOXG1 group. **(J)** Interactions between DEmiRNAs and their target DEPs in the upregulated aging-related pathways in **(H)**. **(K)** Interactions between DEmiRNAs and their target DEPs in the downregulated aging-related pathways in **(I)**.

As key post-transcriptional regulators of gene expression, miRNAs regulate the aging process, and numerous miRNAs are dysregulated in a tissue-specific manner during mammalian aging. Although several miRNAs associated with ARHL have been identified, our understanding of their regulatory mechanisms remains limited. To expand current knowledge beyond previously identified miRNAs, we employed small RNA-seq to explore more changes in miRNAs during the aging process in HEI-OC1 cells and to identify those associated with the transcription factor FOXG1. We first identified DEmiRNAs between the control and D-gal groups, as well as between the control and si-FOXG1 groups. We then extracted the target DEPs corresponding to these DEmiRNAs and performed KEGG pathway analysis of the target DEPs. Finally, we filtered the pathways associated with aging and identified the DEmiRNAs and their corresponding DEPs within these aging-related pathways (Figure 7A). A total of 83 DEmiRNAs were identified between the control and D-gal groups, with 42 upregulated and 41 downregulated DEmiRNAs in the D-gal group (Figure 7B). Figures 7C,D showed the KEGG enrichment analysis of DEPs targeted by upregulated and downregulated DEmiRNAs, respectively. Notably, the targets of upregulated DEmiRNAs were significantly enriched in multiple aging-related pathways, including lysosome; fatty acid degradation; valine, leucine, and isoleucine degradation; glycolysis; gluconeogenesis; lysine degradation; pentose phosphate pathway; and amino sugar and nucleotide sugar metabolism. Meanwhile, the targets of downregulated DEmiRNAs were enriched in pathways associated with aging, such as ribosome biogenesis in eukaryotes, nucleocytoplasmic transport, Hippo signaling, mRNA surveillance, purine metabolism, one-carbon pool by folate, and mitophagy. To identify aging-related molecular targets more precisely, we further extracted specific DEmiRNAs and their corresponding DEPs in these pathways (Figures 7E,F).

We next identified 53 DEmiRNAs between the control and si-FOXG1 groups, comprising 22 upregulated and 31 downregulated miRNAs in the si-FOXG1 group (Figure 7G). KEGG enrichment analysis was conducted on DEPs targeted by the upregulated and downregulated DEmiRNAs. Similarly, multiple age-related pathways were identified among the enriched pathways. The upregulated pathways included fatty acid degradation, valine, leucine, and isoleucine degradation, DNA replication, glycolysis, gluconeogenesis, histidine metabolism, and the p53 signaling pathway (Figure 7H). The downregulated pathways included thermogenesis, oxidative phosphorylation, one-carbon pool by folate, ribosome biogenesis in eukaryotes, glutathione metabolism, and the longevity-regulating pathway (Figure 7I). To further explore miRNAs and proteins potentially associated with FOXG1 regulation, we identified the target DEmiRNAs and their corresponding DEPs within these aging-related pathways, which were presented in Figures 7J,K. These results further indicate that FOXG1 knockdown induces miRNA expression patterns resembling D-gal-induced aging, suggesting that FOXG1 regulates cellular metabolism, mitochondrial function, and redox balance through miRNA-mediated mechanisms.

### 3.8 Metabolomics analysis of the D-gal-induced aging cells

**FIGURE 8:**
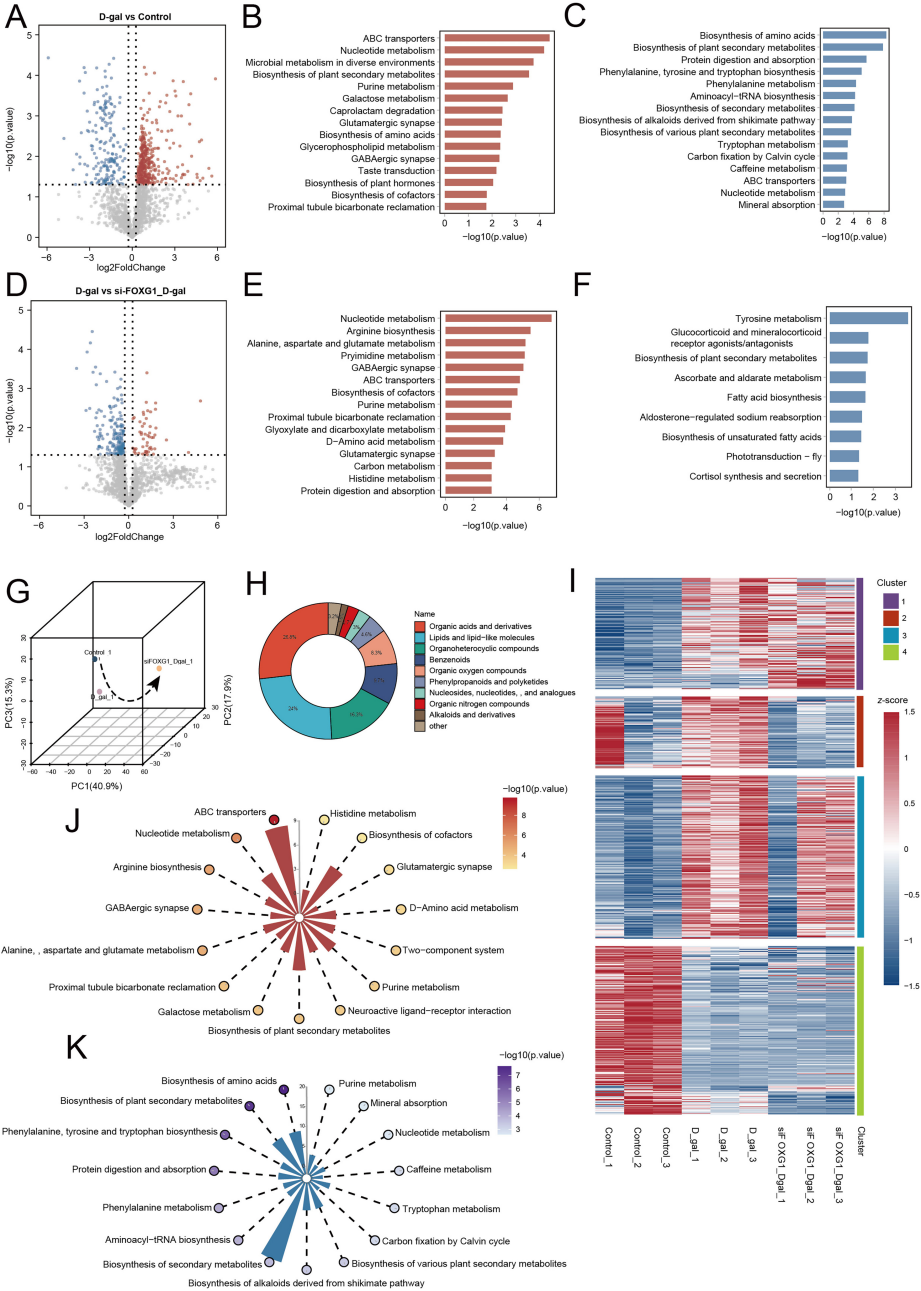
Metabolic alterations in the D-gal-induced aging cells and the D-gal combined with si-FOXG1. **(A)** Volcano plot of differentially expressed metabolites between the control group and the D-gal group (Upregulated metabolites shown as red dots; downregulated metabolites shown as blue dots). **(B)** KEGG enrichment of upregulated metabolites in the D-gal group. **(C)** KEGG enrichment of downregulated metabolites in the D-gal group. **(D)** Volcano plot of differentially expressed metabolites between the D-gal group and the D-gal combined with si-FOXG1 group (Upregulated metabolites shown as red dots; downregulated metabolites shown as blue dots). **(E)** KEGG enrichment of upregulated metabolites in the D-gal combined with si-FOXG1 group. **(F)** KEGG enrichment of downregulated metabolites in the D-gal combined with si-FOXG1 group. **(G)** PCA of metabolomics data from the control group, the D-gal group, and the D-gal combined with si-FOXG group. **(H)** Pie chart of metabolite classes identified in the control group, the D-gal group, and the D-gal combined with si-FOXG1 group. **(I)** K-means clustering analysis of differentially expressed metabolites among the control group, the D-gal group, and the D-gal combined with si-FOXG1 group. **(J)** KEGG enrichment of metabolites in Cluster 1 of **(H)**. **(K)** KEGG enrichment of metabolites in Cluster 4 of **(H)**.

It has been well established that metabolic dysregulation is a hallmark of aging and contributes significantly to the pathogenesis of age-related diseases (Yin et al., 2016; Mattson and Arumugam, 2018). Indeed, both our proteomic and small RNA-seq analyses revealed the disruption of metabolic homeostasis in HEI-OC1 cells subjected to D-gal-induced senescence. To delineate these metabolic alterations in greater depth, we performed untargeted metabolomic analysis on HEI-OC1 cells from three experimental groups: control, D-gal, and D-gal combined with si-FOXG1. PCA results revealed a distinct separation between the D-gal and control groups (Supplementary Figure S5A). Differential analysis identified 509 differentially expressed metabolites (DEMs) between the D-gal and control groups, with 324 upregulated and 185 downregulated metabolites in the D-gal group. The volcano plot and heatmap visually demonstrated the statistical significance of metabolic dysregulation (Figure 8A and Supplementary Figure S5B). To further investigate the biological implications of these metabolites, KEGG pathway enrichment analysis was performed on the upregulated and downregulated DEMs. As shown in Figures 8B,C, ATP-binding cassette (ABC) transporters, purine metabolism, glycerophospholipid metabolism, glutamatergic synapse, and γ-aminobutyric acid (GABAergic) synapse pathways were significantly upregulated in the D-gal group, while amino acid biosynthesis, phenylalanine, tyrosine, and tryptophan biosynthesis, tryptophan metabolism, protein digestion and absorption, and aminoacyl-tRNA biosynthesis were significantly downregulated.

We then analyzed the DEMs between the D-gal and D-gal + si-FOXG1 groups. PCA results revealed a clear separation between these groups (Supplementary Figure S5C). In total, 159 DEMs were identified, with 44 upregulated and 115 downregulated in the D-gal + si-FOXG1 group. As shown in Figure 8D and Supplementary Figure S5D, the expression patterns of these metabolites were visualized using volcano plots and heatmaps. KEGG analysis revealed that the upregulated DEMs highlighted pathways involved in arginine biosynthesis; alanine, aspartate, and glutamate metabolism; ABC transporters; purine metabolism; glutamatergic synapse; and histidine metabolism (Figure 8E). Downregulated metabolites were primarily enriched in tyrosine metabolism, glucocorticoid and mineralocorticoid receptor signaling, ascorbate and aldarate metabolism, fatty acid biosynthesis, unsaturated fatty acid biosynthesis, and cortisol synthesis and secretion (Figure 8F). These results suggested that under D-gal-induced aging conditions, FOXG1 knockdown exacerbates oxidative stress and causes a functional decline in several key protective and regulatory metabolic pathways.

Next, we performed an integrated metabolomic analysis of HEI-OC1 cells from control, D-gal, and D-gal + si-FOXG1 groups. The PCA results showed significant separation among the three groups (Figure 8G). A total of 1,948 metabolites, categorized into 10 chemical classes, were detected (Figure 8H). To elucidate the metabolic mechanisms underlying the exacerbation of aging phenotypes following FOXG1 knockdown, we performed K-means clustering analysis. The 705 DEMs among the control, D-gal, and D-gal + si-FOXG1 groups were stratified into four distinct clusters (Figure 8I). Clusters 1 (*n* = 154 metabolites) and 4 (*n* = 230 metabolites) displayed the most significant trends. The metabolites in Cluster 1 progressively increased from the control group to the D-gal group, with the greatest significant rise in the D-gal + si-FOXG1 group. Conversely, the metabolites in Cluster 4 exhibited a marked decrease in D-gal + si-FOXG1. These two clusters best reflected the metabolic dysregulation exacerbated by FOXG1 knockdown under aging conditions and were thus selected for subsequent KEGG pathway enrichment to clarify their biological significance. KEGG analysis of Cluster 1 metabolites revealed significant enrichment in ABC transporters, nucleotide metabolism, arginine biosynthesis, GABAergic synapses, alanine/aspartate/glutamate metabolism, purine metabolism, glutamatergic synapses, and histidine metabolism (Figure 8J). These pathways are closely associated with neuronal aging and oxidative signaling, indicating a disruption of the excitatory/inhibitory neurotransmission balance in neural networks (Gleichmann et al., 2012; Chen W. et al., 2024). Furthermore, the enrichment of ABC transporters and purine metabolism highlights energy homeostasis impairment and redox stress in HEI-OC1 cells. The metabolites in Cluster 4 were significantly enriched in amino acid biosynthesis; phenylalanine, tyrosine, and tryptophan pathways; protein digestion and absorption; phenylalanine metabolism; tryptophan metabolism; nucleotide metabolism; and purine metabolism (Figure 8K). Significant downregulation of these pathways indicates a functional decline in protein synthesis, amino acid turnover, and mitochondrial energy metabolism in D-gal-induced aging cells, which was further exacerbated by FOXG1 knockdown.

## 4 Discussion

ARHL is a multifactorial disorder arising from the intricate convergence of diverse pathological influences (Jafari et al., 2019). Although pharmacological interventions and gene therapies are being actively investigated to attenuate the progression of ARHL or restore auditory function, the condition remains incurable (Schilder et al., 2019). The D-gal paradigm is extensively employed to generate reliable and reproducible experimental models of accelerated aging (Ho et al., 2003; Li et al., 2016). At elevated concentrations, D-gal is metabolized to galactitol, which accumulates intracellularly, generating aldehydes and hydrogen peroxide, thereby increasing ROS levels (Cuatrecasas and Segal, 1966). In addition, D-gal impairs antioxidant enzyme activity, induces mitochondrial dysfunction, and triggers neurotoxicity and apoptosis (Song et al., 1999; Lei et al., 2008). All these mechanisms are integral to the pathogenesis of ARHL (Woo et al., 2014; Du et al., 2019). Building on our previous studies (Kong et al., 2006; Chen et al., 2010; He et al., 2020), we successfully established both *in vivo* and *in vitro* mimetic aging models using D-gal in C57BL/6J mice and HEI-OC1 cells, establishing the essential experimental platform for the subsequent discoveries.

As a key nuclear transcription regulator, FOXG1 engaged in a multitude of cellular processes across diverse tissues, notably within the brain and the inner ear. It has been reported to be associated with several signaling pathways, including the Wnt, Notch, and bone morphogenetic protein (BMP) signaling pathways (Danesin et al., 2009; Dali et al., 2018; Hettige et al., 2023). In the inner ear, these same pathways execute essential regulatory functions. For instance, previous studies have reported that the knockout of FOXG1 in mouse cochlear HCs resulted in the inhibition of the Wnt signaling pathway, thereby causing HCs apoptosis (He Z. et al., 2019). Basch et al. found that precise regulation of Notch signaling defined the boundaries of the organ of Corti and regulated HC development (Basch et al., 2016). BMP signaling is critically involved in shaping inner ear morphology during development (Ma et al., 2019). Age-related oxidative stress is a well-established central mechanism in ARHL pathogenesis. Mitochondrial impairment drives excessive ROS accumulation, disrupts biological functions, and promotes cellular damage and apoptosis (Xu et al., 2025). FOXG1 emerges as a significant modulator of mitochondrial genomic stability and functional integrity. Abnormal FOXG1 expression impairs mitochondrial energy metabolism, biogenesis, and membrane potential maintenance. Consistent with this role, our experimental data showed a pronounced decrease in FOXG1 expression within the cochlea of D-gal-induced aging mice. In HEI-OC1 cells, low concentrations of D-gal led to increased FOXG1 expression, whereas high concentrations caused a significant decline, consistent with previous findings (He et al., 2020; He et al., 2021). These observations imply that in HEI-OC1 cells, mild D-gal exposure triggers adaptive stress responses, activating FOXG1 and its downstream signaling for cytoprotection. However, when the D-gal concentration exceeded 15 mg/mL, excessive oxidative and inflammatory stress caused severe and irreversible cellular injury, ultimately leading to apoptosis.

Aging is fundamentally defined by a progressive physiological decline in most organs, manifested at the cellular level as structural deterioration and functional impairment (Jin et al., 2024; Palmer et al., 2025). Autophagy, a lysosome-mediated degradation process, constitutes a central pillar of the proteostasis network and is closely linked to cellular quality control. It removes misfolded proteins, pro-inflammatory factors, and dysfunctional organelles to maintain proteostasis and support cellular metabolism (Park et al., 2024). Dysregulation of autophagy affects numerous aging-associated processes at the cellular and molecular levels, including loss of proteostasis, energy imbalance, impaired intercellular communication, and telomere attrition (Kennedy et al., 2014; Kaushik et al., 2021). We found that compared with the control group, the autophagic activity was significantly decreased in the cochlea of D-gal-induced aging mice. In D-gal-treated aging cells, we observed that the expression levels of LC3-II initially increased and then decreased with increasing D-gal concentrations, mirroring changes in FOXG1 expression. These coordinated alterations imply that both autophagy and FOXG1 are jointly engaged in the modulation of the aging process. Additionally, we found that silencing of FOXG1 in cells led to decreased autophagy, whereas overexpression of FOXG1 resulted in increased autophagy. Notably, FOXG1 overexpression partially reversed the decline in autophagy induced by high D-gal concentrations. These findings suggest that FOXG1 modulates the aging process of inner ear HCs by regulating autophagy.

Epigenetic dysregulation, which results in alterations in gene expression, constitutes a fundamental pathophysiological basis of aging and neurodegenerative diseases (de Meireles et al., 2019). H3K9me2 is typically regarded as an inhibitory modification associated with transcriptional silencing (Padeken et al., 2022). Accumulating evidence underscores the critical involvement of H3K9me2 in neurodegenerative diseases. For instance, Zheng et al. observed a significant increase in H3K9me2 levels within the prefrontal cortex of FAD mouse models, a critical cognitive region affected by AD. Consistently, elevated levels of H3K9me2 were detected in the prefrontal cortex of postmortem tissues from patients with AD (Zheng et al., 2019). Recent research has identified upregulated G9a and H3K9me2 in the brains of AD patients. This finding was corroborated by intervention studies in senescence-accelerated mouse-prone 8 (SAMP8) mice, where the G9a inhibitor BIX01294 successfully reversed elevated H3K9me2 levels and ameliorated cognitive deficits (Bellver-Sanchis et al., 2024). In line with its established role in neural aging, our data revealed a marked elevation of H3K9me2 in the cochlea of D-gal-induced aging mice. In the cellular senescence model, H3K9me2 expression initially decreased and then increased with increasing D-gal concentration, exhibiting an inverse trend relative to the expression of FOXG1 and autophagic activity. These findings suggested that H3K9me2 acted as a participant in the aging process. Moreover, inhibiting H3K9me2 with BIX01294 partially rescued the decreased expression of FOXG1 and reduced autophagy in cells exposed to high-dose D-gal. In contrast, FOXG1 knockdown blocked the rescue effects of BIX01294. These results suggest that H3K9me2 contributes to cellular aging by modulating autophagy through FOXG1-dependent signaling.

To delineate the precise mechanisms through which FOXG1 regulates autophagy, we measured the expression of autophagy-linked miRNAs in the cochlea, hippocampus, and cortex of D-gal- treated aging mice. Our analysis revealed a tissue-specific reduction in the levels of miR-34a, miR-96, miR-182, and miR-183 specifically within the aging cochlea, with no significant alterations detected in the hippocampus or cortex. *In vitro* experiments confirmed the downregulation of these miRNAs in D-gal-induced senescent cells, paralleling changes in FOXG1 expression. Knockdown of FOXG1 led to a further decline in the abundance of these miRNAs. Both BIX01294 treatment and FOXG1 overexpression rescued D-gal-induced suppression of these miRNAs. These observations imply that these miRNAs are engaged in the aging process and are likely under the transcriptional influence of FOXG1. In HEI-OC1 cells, inhibition of miR-34a, miR-96, miR-182, and miR-183 expression significantly decreased autophagic activity, elevated ROS accumulation, and increased apoptosis, demonstrating that these miRNAs are closely associated with autophagy and apoptosis. In addition, co-treatment with BIX01294 and miRNA inhibition resulted in decreased autophagy levels, whereas the overexpression of these miRNAs partially restored autophagy under D-gal-induced aging conditions. These results further demonstrate that FOXG1-regulated differential expression of autophagy-related miRNAs functions as an intermediate regulatory axis between H3K9me2 and autophagy. As a transcription factor, FOXG1 binds to the promoter regions of specific miRNAs and activates or inhibits their transcription, which is crucial for their downstream regulatory roles. Weise et al. performed small RNA-seq on 6-week-old *Foxg1^*cre/*+^* and wild-type mice and found that reduced FOXG1 levels were accompanied by decreased precursor miR200a and mature miR-200b/miR-200a/miR429 (Weise et al., 2019). During cranial brain development, *Foxg1^cre–^*mediated conditional deletion resulted in the loss of functional miRNAs, leading to near-complete neuronal loss (Kersigo et al., 2011). Like other gene products, miRNA expression is transcriptionally regulated by transcription factors (Nenna et al., 2022). Xie et al. reported that the transcription factor specificity protein 1 (SP1) influences cochlear apoptosis in an acute hearing loss model by regulating Bcl-2 via miR-204-5p (Xie et al., 2021). Integrating these precedents with our previous findings (Mu et al., 2023), we propose that the transcription factor FOXG1 modulates through the transcriptional control of autophagy-related miRNAs.

To comprehensively understand the pathogenesis of ARHL driven by FOXG1 dysregulation, we employed a multi-omics integrative strategy combining proteomics, small RNA-seq, and metabolomics in the mouse-derived auditory cell line HEI-OC1. Our proteomic analysis identified 1,215 DEPs between the D-gal and control groups (746 upregulated and 469 downregulated), and 941 DEPs between the si-FOXG1 and control groups (649 upregulated and 292 downregulated). The upregulated DEPs in both the D-gal and si-FOXG1 groups were enriched in pathways including fatty acid degradation, peroxisome organization, branched-chain amino acid degradation, glycolysis, gluconeogenesis, lysosome, and lysine degradation, highlighting perturbations in metabolic and redox balance. The downregulated DEPs in both groups were enriched in pathways such as ribosome biogenesis in eukaryotes, one-carbon pool by folate, purine metabolism, nucleocytoplasmic transport, mRNA surveillance, and longevity-regulating pathways. Small RNA-seq identified 83 DEmiRNAs (42 upregulated and 41 downregulated) between the D-gal and control groups, and 53 DEmiRNAs (22 upregulated and 31 downregulated) between the si-FOXG1 and control groups. KEGG analysis of the target DEPs of these miRNAs showed that pathways including fatty acid degradation, branched-chain amino acid degradation, glycolysis, and gluconeogenesis were upregulated in both groups, while pathways related to DNA synthesis and quality control, such as nucleocytoplasmic transport and mRNA surveillance pathway, were downregulated. These findings were consistent with the proteomic results, suggesting that FOXG1 knockdown recapitulates key features of redox imbalance and metabolic remodeling characteristic of aging phenotypes. Among these phenotypes, the most prominent alterations occurred in metabolic remodeling and energy metabolism pathways, which prompted us to conduct further metabolomic analysis. Metabolomic analysis revealed a distinct anabolic-catabolic imbalance in D-gal-induced senescent cells. Pathways associated with degradation and transport (e.g., ABC transporters and purine metabolism) exhibited abnormal activation, whereas core anabolic processes, including amino acid biosynthesis, protein synthesis and absorption, were significantly suppressed. This imbalance contributes to the onset and progression of senescent phenotypes. Moreover, during D-gal-induced aging, FOXG1 knockdown exacerbated oxidative stress, caused a functional decline in a series of protective metabolic pathways, ultimately aggravating aging-related phenotypes. Notably, qPCR validation of the top 4 upregulated and top 4 downregulated overlapping DEPs revealed that Rpl38 exhibited opposite expression trends between the si-FOXG1 and D-gal groups. This observation suggested that while FOXG1 downregulation recapitulated most aging phenotypes, certain proteins might respond differently depending on the upstream stress signal or the degree of metabolic remodeling.

**FIGURE 9:**
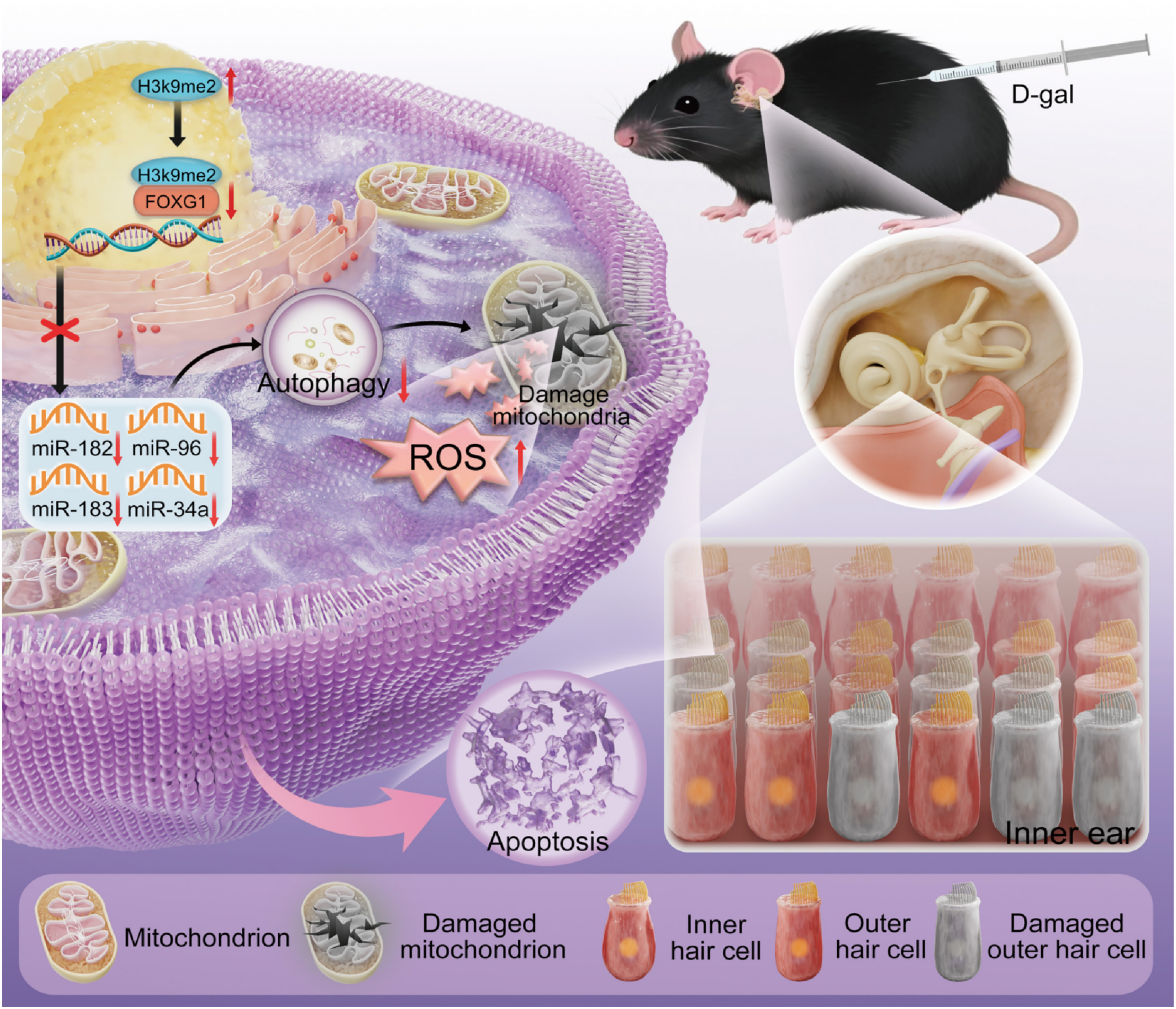
Mechanism of the H3K9me2-FOXG1-miRNAs-autophagy axis in HCs degeneration of ARHL. No AI was used to generate the images in this figure.

In conclusion, our study revealed novel alterations in the H3K9me2-FOXG1-miRNAs-autophagy regulatory axis and its underlying mechanisms during cochlear HCs aging, using D-gal to establish *in vivo* and *in vitro* mimetic aging models. Briefly, during aging, FOXG1 regulates autophagic activity through transcriptional control of autophagy-related miRNAs, thereby modulating ROS levels and apoptosis in HCs. Concurrently, FOXG1 expression is epigenetically repressed by elevated H3K9me2 (Figure 9). Furthermore, multi-omics analysis revealed that FOXG1 knockdown recapitulated and exacerbated key features of redox imbalance and metabolic remodeling characteristic of aging phenotypes. To the best of our knowledge, this work represents the inaugural systematic delineation of the comprehensive regulatory network of FOXG1 in ARHL, providing novel mechanistic insights and promising therapeutic targets for mitigating cochlear aging and oxidative damage.

## Data Availability

The datasets presented in this study can be found in online repositories. The names of the repository/repositories and accession number(s) can be found at: http://www.proteomexchange.org/, PXD072393; https://ngdc.cncb.ac.cn/gsa/s/6pKKG6Mr, CRA035839.
