# Supplementary material for: The H3K9me2-FOXG1-microRNA axis reduces cochlear hair cells damage by modulating autophagy in age-related hearing loss
Source: Front Mol Neurosci. 2026 May 8;19:1834102. doi: 10.3389/fnmol.2026.1834102 (PMC13194610; doi:10.3389/fnmol.2026.1834102)
Supplement: Supplementary file 1 [file Data_Sheet_1.docx]

Supplementary Material

**
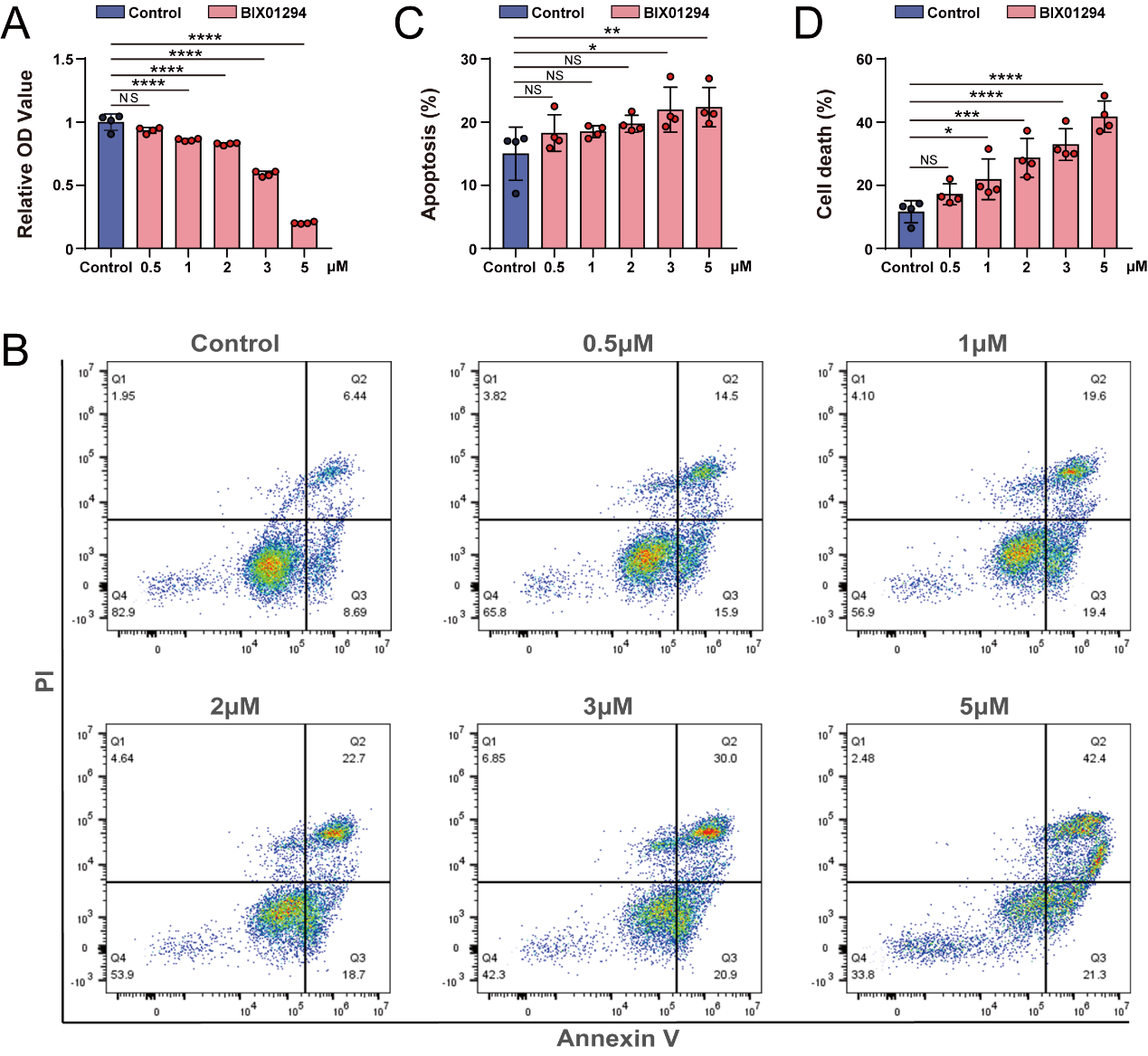
**

**Supplementary Figure 1.** CCK-8 and flow cytometry results for HEI-OC1 cells treated with different concentrations of BIX01294. (A) CCK-8 assay results for HEI-OC1 cells after 24-hour treatment with different concentrations of BIX01294. (B) Flow cytometric analysis of apoptosis in the HEI-OC1 cells treated with different concentrations of BIX01294. (C) Quantification of apoptotic cells in B. (D) Quantification of dead cells in B. Data are presented as mean ± SD; NS = not significant, *p < 0.05, **p < 0.01, ***p < 0.001, ****p < 0.0001.


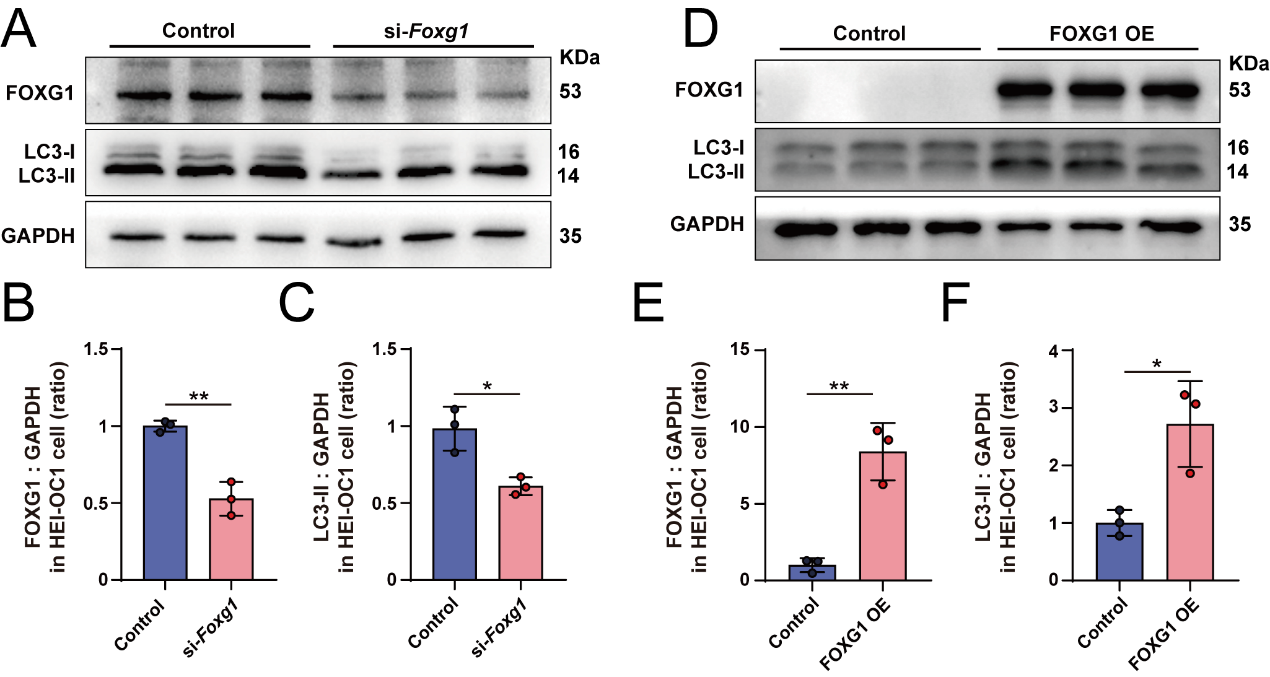


**Supplementary Figure 2.** Western blot analysis of FOXG1 and LC3 expression levels following FOXG1 knockdown and overexpression in HEI-OC1 cells. (A) Western blot of FOXG1 and LC3 expression levels following FOXG1 knockdown in HEI-OC1 cells. (B) Quantitative analysis of the FOXG1 levels in A. (C) Quantitative analysis of the LC3-II levels in A. (D) Western blot of FOXG1 and LC3 expression levels following FOXG1 overexpression in HEI-OC1 cells. (E) Quantitative analysis of the FOXG1 levels in D. (F) Quantitative analysis of the LC3-II levels in D. Data are presented as mean ± SD; *p < 0.05, **p < 0.01.


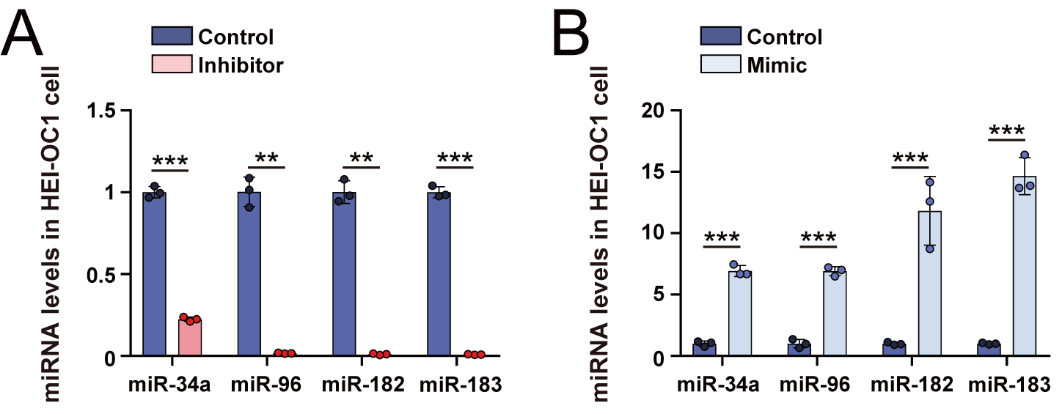


**Supplementary Figure 3.** QRT-PCR results of miR-34a, miR-96, miR-182, and miR-183 expression levels in HEI-OC1 cells following treated with miR-34a, miR-96, miR-182, and miR-183 inhibitors and mimics. (A) qRT-PCR results of miR-34a, miR-96, miR-182, and miR-183 expression levels in HEI-OC1 cells following treated with miR-34a, miR-96, miR-182, and miR-183 inhibitors. (B) qRT-PCR results of miR-34a, miR-96, miR-182, and miR-183 expression levels in HEI-OC1 cells following treated with miR-34a, miR-96, miR-182, and miR-183 mimics. Data are presented as mean ± SD; **p < 0.01, ***p < 0.001.


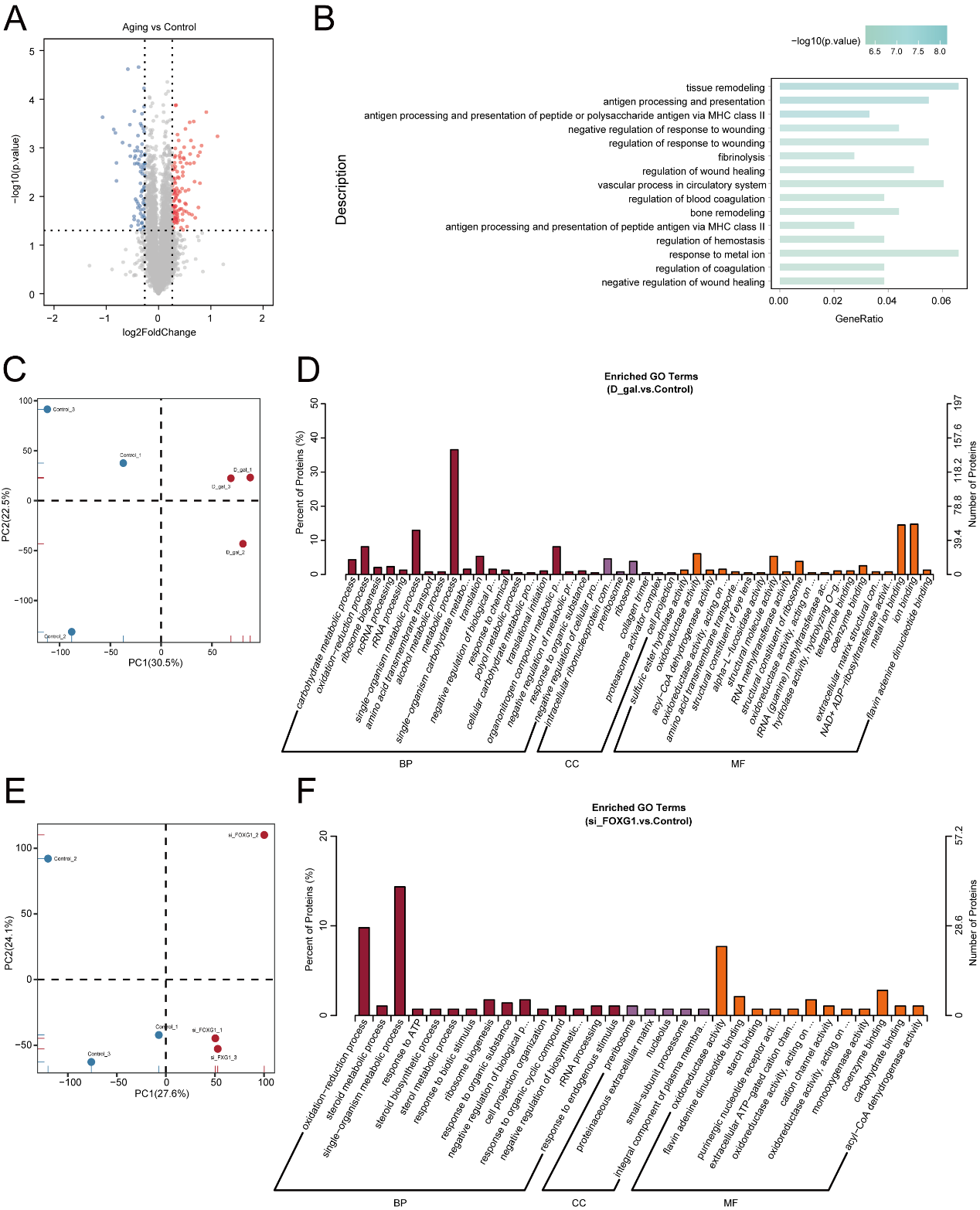


**Supplementary Figure 4.** Proteomics analysis of the aging mouse cochlear and the D-gal-induced aging cells. (A) Volcano plot of DEPs between the control group and the aging group (Up-DEPs shown as red dots; down-DEPs shown as blue dots). (B) GO enrichment analysis of BP for DEPs between the control group and the aging group. (C) PCA of proteomics data from the HEI-OC1 cells in the control group and the D-gal group. (D) GO enrichment analysis of BP, CC and MF for DEPs in HEI-OC1 cells between the control group and the D-gal group. (E) PCA of proteomics data from the HEI-OC1 cells in the control group and the si-FOXG1 group. (F) GO enrichment analysis of BP, CC and MF for DEPs in HEI-OC1 cells between the control group and the si-FOXG1 group.


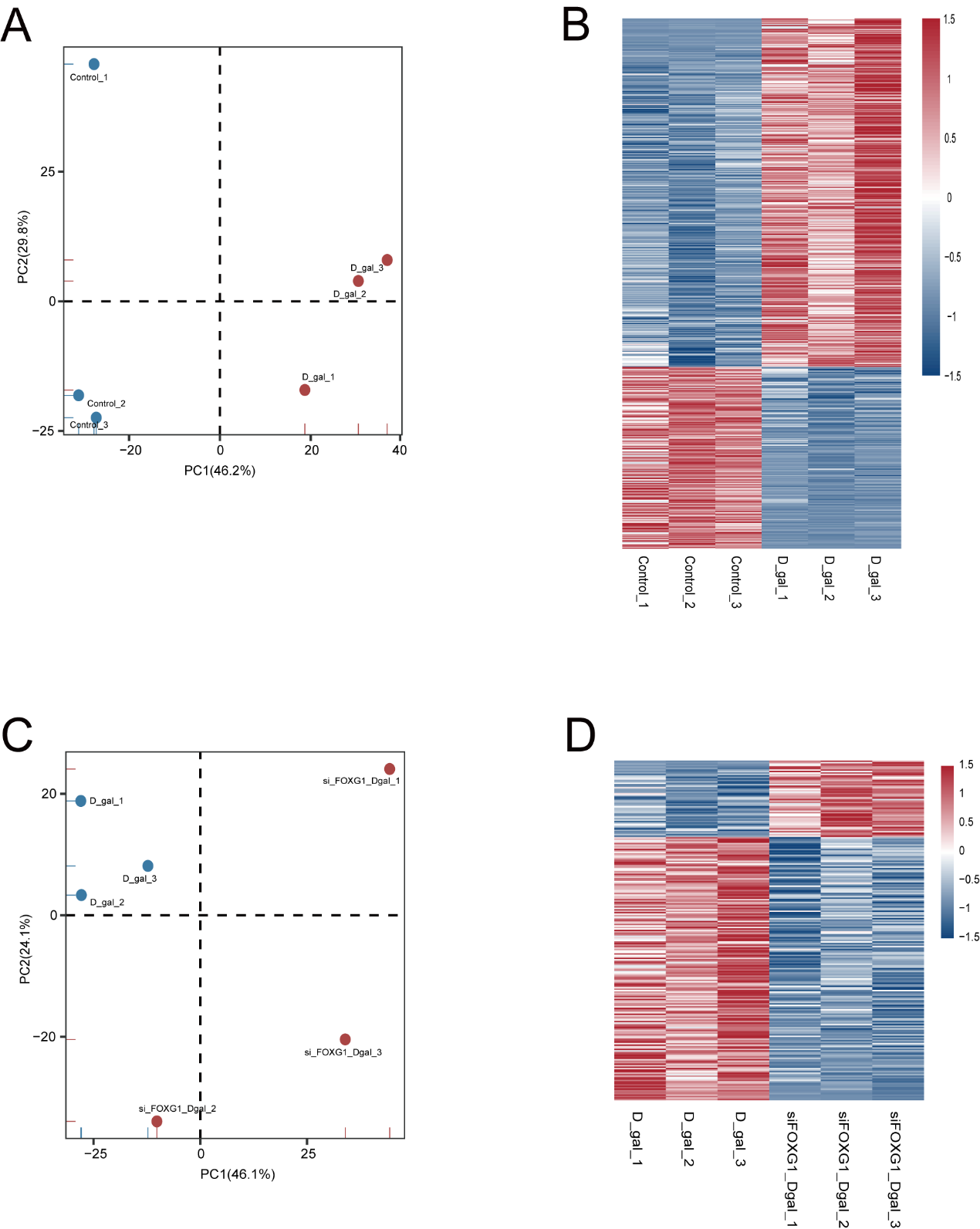


**Supplementary Figure 5.** Metabolomics analysis of HEI-OC1 cells in the D-gal group and the D-gal combined with si-FOXG1 group. (A) PCA of metabolomics data from the HEI-OC1 cells in the control group and the D-gal group. (B) Heatmap of differentially expressed metabolites in HEI-OC1 cells between the control group and the D-gal group. (C) PCA of metabolomics data from the HEI-OC1 cells in the D-gal group and the D-gal combined with si-FOXG1 group. (D) Heatmap of differentially expressed metabolites in HEI-OC1 cells between the D-gal group and the D-gal combined with si-FOXG1 group.

**Table S1:** Primer sequences for qRT-PCR

| Gene |  | Primers |
| --- | --- | --- |
| *Trex1* | Forward | 5’-TGCCTGCTTCTCGGTTCAG-3’ |
|  | Reverse | 5’-CCAGTGGCTTCCAGGTCTAA-3’ |
| *Irf9* | Forward | 5’-GGGGTATGGTAAGGAGAAGGATG-3’ |
|  | Reverse | 5’-AAATGGCCACTCTCCACCTG-3’ |
| *Aqp1* | Forward | 5’-CCGAGACTTAGGTGGCTCAG-3’ |
|  | Reverse | 5’-CAGTGTAGTCAATCGCCAGC-3’ |
| *Hspb1* | Forward | 5’-TCACCCGGAAATACACGCTC-3’ |
|  | Reverse | 5’-GGCCTCGAAAGTAACCGGAA-3’ |
| *Rpl38* | Forward | 5’-TTCCCCGTTCTCTTCGGTTC-3’ |
|  | Reverse | 5’-AATTTTCCGAGGCATGGCGA-3’ |
| *Rbis* | Forward | 5’-CGCGTGAACTTTGACAATGGC-3’ |
|  | Reverse | 5’-CTGGTTTTGCCTTGTTTTTAGCC-3’ |
| *Ybx3* | Forward | 5’-GAAGCAGCAAACGTGACTGG-3’ |
|  | Reverse | 5’-CAATCTCACCAGCATTACGGG-3’ |
| *Esf1* | Forward | 5’-CGGGCTCTCCTTGGGATTTTATT-3’ |
|  | Reverse | 5’-CTGAACCGCTGGTCATCCAT-3’ |
| *Gapdh* | Forward | 5’-AGGTCGGTGTGAACGGATTTG-3’ |
|  | Reverse | 5’-TGTAGACCATGTAGTTGAGGTCA-3’ |
